# A Pool of Eight Virally Vectored African Swine Fever Antigens Protect Pigs against Fatal Disease

**DOI:** 10.3390/vaccines8020234

**Published:** 2020-05-18

**Authors:** Lynnette C. Goatley, Ana Luisa Reis, Raquel Portugal, Hannah Goldswain, Gareth L. Shimmon, Zoe Hargreaves, Chak-Sum Ho, María Montoya, Pedro J. Sánchez-Cordón, Geraldine Taylor, Linda K. Dixon, Christopher L. Netherton

**Affiliations:** 1 The Pirbright Institute, Ash Road, Pirbright, Surrey GU24 0NF, UK; lynnette.goatley@pirbright.ac.uk (L.C.G.); ana.reis@pirbright.ac.uk (A.L.R.); raquel.portugal@pirbright.ac.uk (R.P.); hannah.gold@outlook.com (H.G.); gareth.shimmon@gmail.com (G.L.S.); zoe.hargreaves@pirbright.ac.uk (Z.H.); maria.montoya@cib.csic.es (M.M.); Pedro.Sanchez-Cordon@apha.gov.uk (P.J.S.-C.); geraldine.taylor@pirbright.ac.uk (G.T.); linda.dixon@pirbright.ac.uk (L.K.D.); 2 Gift of Hope Organ and Tissue Donor Network, Itasca, IL 60143, USA; sho@giftofhope.org

**Keywords:** African swine fever virus, viral-vector, adenovirus, modified vaccinia Ankara (MVA), vaccine, pathology

## Abstract

Classical approaches to African swine fever virus (ASFV) vaccine development have not been successful; inactivated virus does not provide protection and use of live attenuated viruses generated by passage in tissue culture had a poor safety profile. Current African swine fever (ASF) vaccine research focuses on the development of modified live viruses by targeted gene deletion or subunit vaccines. The latter approach would be differentiation of vaccinated from infected animals (DIVA)-compliant, but information on which viral proteins to include in a subunit vaccine is lacking. Our previous work used DNA-prime/vaccinia-virus boost to screen 40 ASFV genes for immunogenicity, however this immunization regime did not protect animals after challenge. Here we describe the induction of both antigen and ASFV-specific antibody and cellular immune responses by different viral-vectored pools of antigens selected based on their immunogenicity in pigs. Immunization with one of these pools, comprising eight viral-vectored ASFV genes, protected 100% of pigs from fatal disease after challenge with a normally lethal dose of virulent ASFV. This data provide the basis for the further development of a subunit vaccine against this devastating disease.

## 1. Introduction

African swine fever virus (ASFV) is the etiological agent of a disease of domestic pigs and wild boar that is typically fatal and for which there is no vaccine or prophylactic. The disease was first reported in the early 20th century and is considered endemic in sub-Saharan Africa. ASFV first appeared in Europe in 1957 and again in 1960 in Portugal wherefrom the virus became established in the Iberian Peninsula and from there caused outbreaks across Western Europe as well as in the Caribbean and Brazil. Early experiments showed that passage of the virus through tissue culture led to its attenuation and that pigs immunized with these attenuated viruses were protected from acute disease after challenge with homologous strains. Such attenuated viruses were used in the Iberian Peninsula in the early 1960s, however their efficacy in the field was not satisfactory and induced a chronic form of the disease [1,2,3]. With the exception of the island of Sardinia, ASF was eradicated outside of Africa by the mid-1990s, however it returned to Georgia in 2007 and has since spread throughout Eastern Europe and Eastern Asia. Millions of animals have been killed by the virus or destroyed in attempts to control the disease.

Current approaches to ASF vaccine design concentrate on rational attenuation of virulent viruses by targeted gene deletion [4,5,6,7,8,9,10] or development of subunit vaccines. The latter approach has been limited by the choice of a delivery system and knowledge of protective antigens [11]. To date, protein, DNA and viral vectored ASFV subunit vaccines have been tested in challenge studies. Recombinant p30, p72, p54 and p22 (encoded by the *CP204L*, *B646L*, *E183L* and *KP177R* genes respectively) induced neutralizing antibodies in pigs, but did not protect the animals from fatal disease [12]. However, an *E183L*-*CP204L* fusion protein also induced neutralizing antibodies in pigs and did protect the animals from severe disease after challenge [13]. Recombinant CD2v (*EP402R*) induced an antibody response in pigs capable of inhibiting hemadsorption of red blood cells to ASFV-infected macrophages and protected two out of three animals from fatal disease after challenge with virulent ASFV [14]. A DNA vaccination approach using a fusion of the E183L, CP204L and the extracellular domain of *EP402R* did not protect animals unless they were also fused to ubiquitin [15]. This approach has been extended to immunization with a library of ASFV genomic DNA fragments, which protected 60% of pigs from a fatal disease [16]. Interestingly a combinatorial approach of DNA-prime followed by protein-boost that included *EP402R*, *CP204L* and *E183L* did not protect animals from fatal disease despite inducing antigen-specific responses [17].

We and others have shown the utility of viral vectors as a route to express ASFV antigens in pigs and induce antigen-specific antibody and cellular immune responses [18,19,20,21,22,23]. A pool comprised of the ASFV genes *A151R*, *B646L* (p72), *C129R*, *CP204L* (p30), *CP530R* (pp62), *E146L*, *I73R*, *I125L*, *L8L*, *M448R*, *MGF110-4L* and *MGF110-5L* vectored by replication-deficient human adenovirus 5 (rAd) prime and modified vaccinia Ankara (MVA) boost led to reduced clinical signs and reduced levels of viremia in a proportion of pigs after challenge with the virulent OUR T88/1 isolate [21]. *B646L* (p72), *CP204L* (p30), *CP530R* (pp62), *E183L* (p54) in combination with the mature p37 product and two sections of the mature p150 protein of the pp220 polyprotein (*CP2475L* gene) have been delivered to pigs using an rAd homologous prime and boost strategy with two different adjuvants [18,22]. After challenge with the Georgia 2007/1 isolate, 55% of the animals in one of the adjuvanted groups survived to the end of the experiment, however, interpretation of this result is complicated by the observation that the challenge model did not result in clinical signs typical of acute ASF in all of the controls [22]. One complicating factor in designing subunit vaccines to ASFV is that certain antigens, or combinations of antigens, can induce enhanced disease in swine [15,21,22]. The mechanism(s) and the individual antigens responsible for the vaccine-induced enhanced disease are unclear, but DNA vaccination experiments showed a clear link between the induction of antibodies to the CP204L, E183L and EP402R fusion protein and enhanced disease [15]. However, experiments with live attenuated viruses suggest a role for both humoral [24,25] and cellular [26,27,28] responses in protection against virulent strains of ASFV and therefore a subunit vaccine may need to induce both arms of the immune response for effective protection.

Taken together, these results suggest that a viral-vectored vaccine against ASF is a feasible approach, however, the choice of antigens is key to success. In a previous study, we immunized domestic pigs with pools of ASF antigens using a DNA-prime/vaccinia virus-boost regime and identified antibody responses by ELISA and cellular responses by ELIspot using recombinant protein as recall antigens. The DNA-prime/vaccinia virus-boost approach identified a novel subset of immunogenic antigens, but the immunization regime did not affect the clinical progression of disease after challenge, although reduced viremia was seen three days post-infection and a reduced viral load in some tissues was observed at termination [29]. We, therefore, decided to refine our approach, using rAd prime and MVA boost as a delivery system with the antigens shown as immunogenic *ex-ASFV*. Here we show that a combination of eight different antigens can protect pigs from fatal disease after challenge with a virulent genotype I strain of ASFV.

## 2. Materials and Methods 

### 2.1. Antibodies

Monoclonal antibody C18 against *CP204L* (p30) has been described previously [30]. Rat anti-HA monoclonal antibody 3F10 was purchased from Roche, mouse anti-V5 monoclonal antibody SV5-PK1 was from BioRad (CA, USA), and mouse anti-porcine IFNγ monoclonal antibodies P2F6 and P2C11 were from Thermo-Fisher Scientific.

### 2.2. Viruses and Cells

Tissue cultured adapted Ba71v and virulent OUR T88/1 ASFV strains have been described previously [31,32]. OUR T88/1 was grown and titrated on bone marrow cells prepared from the femurs of four to six-week-old Large White outbred pigs, Ba71v was grown on Vero cells. Bone marrow cells were cultured for 3 days in EBSS (Sigma-Aldrich, Gillingham, UK) supplemented with 4 mM HEPES, 10% heat-inactivated porcine serum (BioSera) and 100 IU/mL penicillin and 100 µg/mL streptomycin (Sigma-Aldrich, Gillingham, UK) in plastic multi-well plates or culture flasks prior to infection. Vero cells were maintained in DMEM-HEPES supplemented with 10% heat-inactivated foetal calf serum and 100 IU/mL penicillin and 100 µg/mL streptomycin. A mock inoculum was prepared from uninfected cell cultures. Infectious virus titres were determined by end-point dilution using the Spearman–Karber method as the amount of virus causing hemadsorption in 50% of infected cultures (HAD) or as the amount of virus causing 50% of cells staining positive for ASFV early protein p30 by immunofluorescence (infectious units (IU). PBMC were cultured in RMPI GlutaMAX (Thermo Fisher, Hemel Hempstead,UK), 25 mM HEPES supplemented with 10% fetal calf serum, 1 mM sodium pyruvate, 50 µM 2-mercaptoethanol, 100 IU/mL penicillin and 100 µg/mL streptomycin (RPMI/10). ASFV genomic DNA was detected using real-time quantitative polymerase chain reaction (qPCR) as previously described [33]. Nucleic acid was extracted in duplicate using the MagMax Universal Extraction kit (Thermo Fisher, Hemel Hempstead,UK), and each extraction subject to duplicate qPCR. Data points represent the mean of these duplicate qPCR. For experiment 2, virus antigen was detected in the bloodstream five days post-infection using ASFV-specific lateral flow devices (INgezim ASF CROM Ag, Ingenasa, Madrid, Spain).

### 2.3. Recombinant Vectors

Vectors expressing influenza NP, *B646L* (p72) and *CP204L* (p30) have been described previously [21,34]. ASFV open reading frames (ORFs) were codon-optimized for expression in *Sus scrofa*, synthesized and cloned into pcDNA3.1zeo(+) (Thermo Fisher, Hemel Hempstead,UK). Complete ORFs were synthesized with an HA tag at the 3′-end. Gene sequences were derived from the genotype I OUR T88/3 isolate of ASFV with the exception of the *EP153R* and *MGF360-11L* genes which were from the genotype I Benin 1997/1 isolate [35]. ASFV ORFs were then sub-cloned into transfer plasmids for making recombinant replication-deficient human adenovirus 5 (rAd) and recombinant modified vaccinia Ankara (MVA) using standard techniques. Purified viral vectors were generated by the Jenner Institute Viral Vector Core Facility (Oxford, UK) [36,37]. The MVA transfer plasmids, and hence the final viral vectors, also contained GFP under the control of a viral promoter to enable rapid plaque purification of positive clones. All the vaccine vectors expressed protein products of the expected sizes (Figures S1–S3) including proteins consistent with the glycosylated forms of *EP153R* and *I329L.*

A plasmid containing the V5 epitope sequence was created by ligating overlapping oligonucleotides into restriction endonuclease digested pcDNA3.1zeo (+). Codon-optimized ASFV genes *E199L, EP153R, EP364R, F317L, I329L, MGF360-11L*, *MGF505-4R* and *MGF505-5R* were then amplified by PCR and sub-cloned in frame with the V5 epitope to create C-terminally tagged expression plasmids and confirmed by sequencing.

### 2.4. Swine Leucocyte Antigen (SLA) Genotyping

Genotyping of three *SLA* class I (SLA-1, SLA-2, SLA-3) and three class II (DRB1, DQB1, DQA) genes was performed using low-resolution PCR-based assays with sequence-specific typing primers as previously described [38,39]. Modifications were made to the typing primer panels to broaden the allele coverage with the increasing number of *SLA* alleles. *SLA* haplotypes were deduced based on comparison with published haplotypes [38,40,41,42].

### 2.5. Animal Experiments and Ethics Statement

All of the animal experiments were carried out under the Home Office Animals (Scientific Procedures) Act (1986) (ASPA) and were approved by the Animal Welfare and Ethical Review Board (AWERB) of The Pirbright Institute and the AWERB of the Animal and Plant Health Agency (APHA), Weybridge. Prime and boost with viral vectors were carried out at APHA. The animals were housed in accordance with the Code of Practice for the Housing and Care of Animals Bred, Supplied or Used for Scientific Purposes, and bedding and species-specific enrichment were provided throughout the study to ensure high standards of welfare. Through careful monitoring, pigs that reached the scientific or humane endpoints of the studies were euthanized by an overdose of anesthetic (see Supplementary data). All procedures were conducted by Personal License holders who were trained and competent and under the auspices of Project License PPL70/8852.

Female Landrace × Large white pigs were obtained from a high health farm in the UK and randomly assigned to each group prior to immunization. Scoring of clinical signs and macroscopic lesions assessed at post-mortem were as described previously [27,43]. 

#### 2.5.1. Experiment 1

Groups of six, eight-week-old, outbred pigs were inoculated intramuscularly in the neck with rAd expressing the influenza nucleoprotein (PR8 strain) (Control, pigs 270 to 275), a pool of eight rAd expressing ASFV ORFs (Antigen Pool A, pigs 264 to 269) or a pool of eight different rAd expressing ASFV ORFs (Antigen Pool B, pigs 276 to 281) (Table 1). Each rAd expressing ASFV genes was administered at a dose of 5 × 10 ^9^ IU, and the controls were immunized with an equivalent total dose of control rAd, therefore all animals were primed with a total of 4 × 10^10^ IU rAd per pig. Four weeks later, the pigs were inoculated in the same site with MVAs expressing the same ORFs with the exception of *EP364R*, *I329L*, *MGF505-4R* and *MGF505-5R* where rAd was used instead as MVAs expressing these genes were not available. Each MVA expressing an ASFV gene was administered at a dose of 7.5 × 10^7^ pfu and each rAd at a dose of 5 × 10^9^ IU. Animals in the control group were given the equivalent of seven doses of MVA and two of rAd. Therefore, the Control group was boosted with a total of 3.8 × 10^8^ pfu MVA and 2 × 10^10^ IU rAd, Antigen Pool A with a total of 5.25 × 10^8^ pfu MVA and 5 × 10^9^ IU rAd and animals given Antigen Pool B a total of 3 × 10^8^ pfu of MVA and 2 × 10^10^ IU rAd per pig. Four weeks later, the animals were challenged by the intramuscular route in the rump with 10,000 HAD OUR T88/1. Whole blood and heparinized blood was collected before each immunization (Days 0 and 28) and two weeks after each immunization (Days 14 and 42). Blood was also collected before (Day 59) and seven days after challenge (Day 66) with ASFV.

#### 2.5.2. Experiment 2

Groups of six, eight-week-old, outbred pigs were inoculated intramuscularly in the neck with rAd expressing the influenza nucleoprotein (PR8 strain) (Control, pigs 455 to 460), a pool of eight rAd expressing ASFV ORF (Antigen Pool A, pigs 461 to 466) or a pool of five rAd expressing ASFV ORFs (Antigen Pool C, pigs 449 to 454) (Table 1). Each rAd was administered at a dose of 1.5 × 10^10^ IU and the controls were immunized with an equivalent total dose. Therefore, animals are given the control antigen and Antigen Pool A were primed with a total of 1.2 × 10^11^ IU rAd per pig and those given Antigen Pool C a total of 7.5 × 10^10^. Four weeks later, the pigs were inoculated in the same site with MVAs expressing the same ORFs with the exception of *MGF505-5R* where rAd were used instead.

Each MVA expressing an ASFV gene was administered at a dose of 2 × 10^8^ pfu and each rAd at a dose of 1.5 × 10^10^ IU. Animals given NP Control were boosted with a total of 1.2 × 10^9^ pfu MVA, Antigen Pool A with a total of 1.4 × 10^9^ pfu MVA and 1.5 × 10^10^ IU rAd and animals given Antigen Pool C a total of 8 × 10^8^ pfu of MVA and 1.5 × 10^10^ IU rAd per pig. Four weeks later the animals were challenged by the intramuscular route in the rump with 10,000 HAD OUR T88/1. Whole blood and heparinized blood was collected before each immunization (Days 0 and 28) and two weeks after each immunization (Days 14 and 42). Blood was also collected before (Day 59) and seven days after challenge (Day 66) with ASFV.

### 2.6. Interferon Gamma (IFNγ) ELISpot

Peripheral blood mononuclear cells (PBMC) were purified from heparinized blood using histopaque (Sigma-Aldrich, Gillingham, UK) gradients and then washed extensively with PBS. The response to ASFV was analyzed after in vitro stimulation of fresh cells, however, the response to peptides was analyzed using cells that had been frozen, where viability was ≥90% after thawing. Only samples from animals for which there was sufficient material to test antigen-specific responses at all relevant time points were analyzed in this way. PVDF membrane multiwell plates (Millipore, Abingdon, UK. MAIPS4510) were coated overnight at 4 °C with 4 µg/mL anti-porcine IFNγ (P2F6) in 0.5 M carbonate-bicarbonate coating buffer and then washed with PBS. Cells were plated in duplicate at two different dilutions, typically 5 × 10^5^ and 2.5 × 10^5^ per well in RMPI/10. Cells were then incubated overnight in a final volume of 200 µL with media alone, 0.5% DMSO, 10^5^ HAD of OUR T88/1 or an equivalent volume of mock inoculum, or 2.5 µg/mL PHA, or peptide pools. Twenty-mer peptides overlapping by 10 amino acids were supplied at 1 to 3 mg scale (Mimotopes). The maximum amount of peptides in any one pool was 24 and therefore there was at most 72 µg of peptides per well, with each individual peptide being at a final concentration of 5 to 15 µg/mL. The molecular mass of the peptides varied between 1737.86 and 2758.11, therefore the final molarity of the peptides varied between 1.8 and 8.6 µM. Cells were lysed by incubating for 5 min in water and then washed with PBS. Biotinylated anti-porcine IFNγ (P2C11), followed by streptavidin-conjugated to alkaline phosphatase, and then AP Conjugate Substrate Kit (Bio Rad, Kidlington, UK) was used to visualize spots, which were then counted using an ELIspot Reader System (Autoimmun Diagnostika, Strasberg, Germany). The number of spots was converted into the number of spots per million cells and the mean of duplicate wells plotted. In experiments where the number of IFNγ secreting cells was measured over time, the response to the background (the highest of media/mock/DMSO) was subtracted from the response to whole virus or peptide—this is indicated in the figure legends.

### 2.7. Fixed-Cell Assays

#### 2.7.1. Immunoperoxidase Assay (IPA) against the Whole Virus

Anti-ASFV antibody titres were determined using an immunoperoxidase assay, by incubating two-fold serial dilutions of sera on Ba71v-infected Vero cells fixed 16 h post-infection with 4% paraformaldehyde [44]. Cells were permeabilized with 0.2% Triton X-100, blocked with 5% milk in PBS-0.05% Tween 20 for one hour, then incubated with diluted sera for another hour and finally with HRP-protein A conjugate (Thermo Fisher, Hemel Hempstead, UK). Cells were washed five times with PBS 0.05% Tween20 between each step. Positive wells were identified by AEC staining (2 mM 3-amino-9-ethylcarbazole and 0.015% H_2_O2 (Sigma-Aldrich, Gillingham, UK) diluted in 50 mM sodium acetate buffer). All pigs showed non-specific background staining of uninfected cells after incubating with day 0 sera at dilutions varying from 1:16 to 1:128. 

#### 2.7.2. Immunofluorescence Assay (IFA) against Individual Proteins

The presence of antibodies to individual proteins was determined using indirect immunofluorescence. Sera were used at a single dilution of 1:100 and incubated with Vero cells transfected with each of the individual genes fused to a V5 tag. Pre-immunization and pre-challenge sera were tested from pigs immunized with the pools of viral vectors expressing ASFV ORFs. Only pre-challenge sera from pigs immunized with the control antigens were analyzed for antibodies to ASFV proteins. Mouse anti-V5 tag (AbD Serotec, Oxford, UK. MCA1360) diluted 1:1000 was used as a positive control for V5 fusion gene expression. In indirect immunofluorescence, the anti-V5 antibody was incubated simultaneously with the animal sera on transfected cells. Secondary antibodies were goat anti-mouse IgG (H+L)-*AF594* (Thermo Fisher, Hemel Hempstead, UK) and goat anti-porcine IgG (H+L)-AF488 (Southern Biotech, Birmingham, UK), all added at a dilution of 1:1000 to the cells. The cells were then observed under the fluorescence microscope and screened for simultaneous green and red fluorescence, resulting from recognition by specific antibodies in the pig serum of the expressed viral protein and the presence of the V5 tag respectively.

### 2.8. Indirect ELISA

*CP204L*, *B602L* and *B646L* indirect ELISAs were carried out as previously described [45]. Maxisorp ELISA plates (Nunc) were coated with ASFV recombinant proteins (50 μL per well) diluted (1–10 μg/mL) in coating buffer (50 mM sodium carbonate/bicarbonate buffer, pH 9.6) and incubated overnight at 4 °C. The wells were then washed three times with PBS plus 0.05% Tween 20 and blocked with PBS plus 5% milk (200 μL per well) at 37 °C for 1 h. After blocking, plates were washed five times as above and incubated for 1 h at 37 °C with pig sera diluted 1:100 in PBS plus 5% milk (50 μL per well). The plates were again washed five times and incubated with protein A–horseradish peroxidase (Pierce) diluted 1:5000 (100 μL per well) for 1 h at 37 °C. Finally, plates were washed again and developed with 3-dimethylaminobenzoic acid/3-methyl-2-benzothiazolinone hydrazine hydrochloride/H_2_O_2_ dissolved in 0.1 M phosphate buffer. After stopping the reaction with 3 M H_2_SO_4_ (50 μL per well), the absorbance at 620 nm was read on a Cytation3 microplate reader (Biotek, Swindon, UK).

### 2.9. Statistical Analysis

Statistical analysis was performed with GraphPad Prism 8 (GraphPad Software, San Diego, CA, USA). Unless stated otherwise two-way repeated measures ANOVA with the Geisser–Greenhouse correction and Tukey’s multiple comparison test was used to compare data between and within groups of animals. F ratios are reported in form F (dfn, dfd) = x, where dfn indicates the degrees of freedom for the numerator and dfd the degrees of freedom of the denominator.

## 3. Results

### 3.1. Pools of Eight Viral Vectors Induce ASFV-Specific and Antigen-Specific Responses in Swine

**Figure 1:**
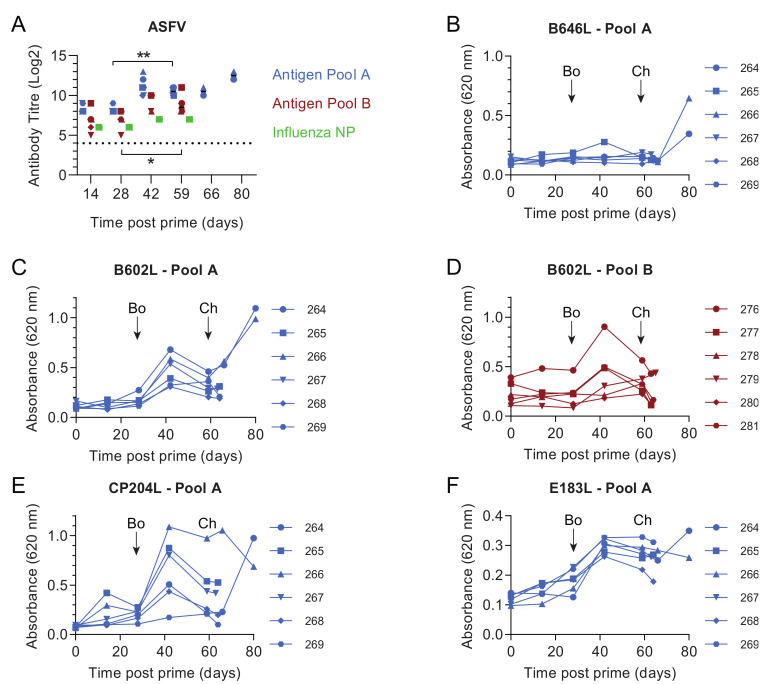
Antibody responses in pigs from experiment 1 primed with rAd encoding Antigen Pool A (blue), Antigen Pool B (red) or influenza NP (green). Animals were boosted with rAd/MVA 28 days after priming and challenged with ASFV 59 days after priming. (**A**) The titer of anti-ASFV antibodies in the serum of the indicated animals was determined by immunoperoxidase assay. Antibodies to ASFV proteins *B646L* (**B**), *B602L* (**C**, **D**), *CP204L* (**E**) and *E183L* (**F**) were detected in 1:100 dilutions of serum collected from the indicated animals at the indicated times post-prime by ELISA, and expressed as the absorbable at 620 nm. Timing of boost (Bo) and challenge (Ch) are indicated on panels B to F. Differences between antibody titres were assessed by repeated-measures two-way ANOVA,* *p* ≤ 0.05, ** *p* ≤ 0.01.

In experiment 1 two groups of six outbred pigs were primed with two separate pools of eight replication-deficient human adenovirus 5 (rAd) vectors expressing individual ASFV genes and another group of six pigs was immunized with rAd expressing the influenza virus nucleoprotein as a control. Antigen Pool A contained the ASFV genes *B602L*, *B646L* (p72), *CP204L* (p30), *E183L* (p54), *E199L*, *EP153R* (C-type lectin), *F317L* and *MGF505-5R*, whereas Antigen Pool B contained the genes B*602L, EP153R, EP364R, F317L, I329L, MGF360-11L, MGF505-4R* and *MGF505-5R* (Table 1). Following on from our previous work, gene sequences were derived from the genotype I ASFV isolate OUR T88/3 as immunization with this virus can protect pigs from OUR T88/1 and Benin 1997/1. Since *EP153R* and *MGF360-11L* are either deleted or truncated in the OUR T88/3 genome sequences for these two genes were derived from the closely related genotype I isolate Benin 97/1. Four weeks later, the animals were boosted with the same pools of antigens vectored using rAd or modified vaccinia Ankara (MVA). Approximately four weeks post-boost (59 days post-prime), the animals were challenged with the virulent OUR T88/1 strain of ASFV. Immunization with both pools of ASFV antigens induced antibodies that reacted with ASFV-infected cells (Figure 1A). Non-specific staining of Vero cells was detected in pre-immune sera from all of the pigs but significant differences from the background were induced by Antigen Pool A at all times points post-prime (*p* ≤ 0.0008, F(15, 60) = 4.302) and by Antigen Pool B after boost (*p* ≤ 0.0273, F(15, 60) = 4.302). Antibody titers against infected cells were higher pre-challenge than pre-boost in both groups of pigs (Pool A, *p* = 0.0047, Pool B; *p* = 0.0303; F (15, 60) = 4.302) suggesting that the boost may have increased the level of ASFV-specific antibodies in circulation, however, there was no difference in titers between the two groups of pigs. Indirect ELISAs were used to detect antibodies against *B602L*, *CP204L*, *E183L* and *IFA* to detect antibodies against *F317L*, *EP364R* and *I329L* in serum from different animals in the two groups (Figure 1B–F; Table 2), although antibodies to *EP364R* were only found in one animal. No antibodies were detected to *B646L* by indirect ELISA or to *EP153R*, *MGF360-11L*, *MGF505-4R* or *MGF505-5R* by IFA.

**Figure 2:**
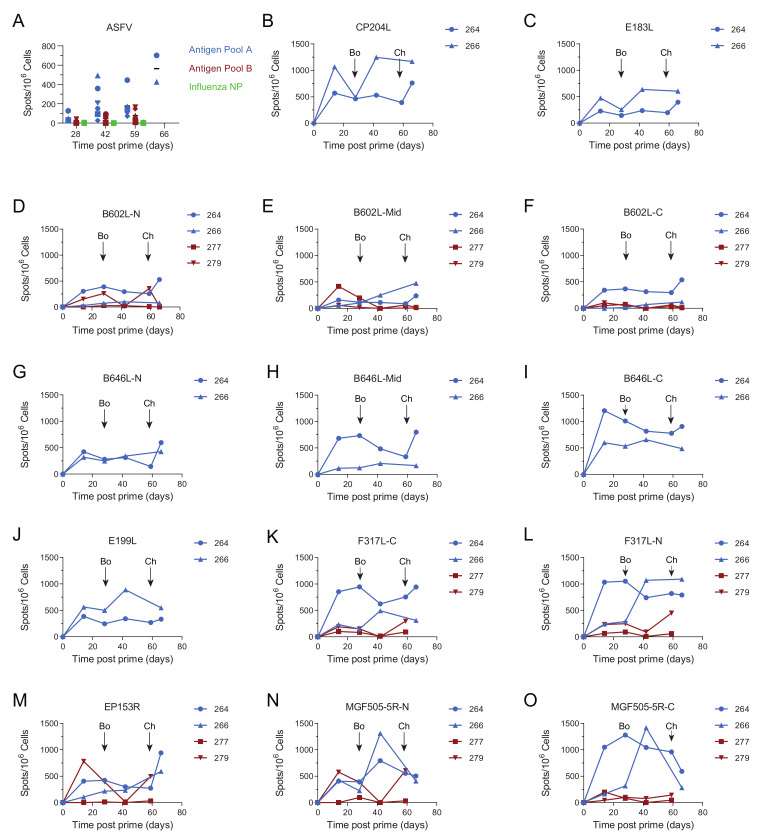
Virus and antigen-specific IFNγ responses in animals from experiment 1 immunized with Antigen Pool A (blue), Antigen Pool B (red) or Influenza NP (green). (**A**) The number of ASFV-specific IFNγ producing peripheral blood mononuclear cells (PBMC) were enumerated by ELIspot, on the indicated days post-prime, following in vitro stimulation with ASFV or peptides. Data points indicate the number of spots per million cells induced by the whole virus after background subtraction. (**B**–**O**). The number of IFNγ-producing PBMC stimulated by pools of peptides was enumerated by ELIspot on the indicated days post-prime. N, mid and C indicate pools of peptides that correspond to the N-terminus, mid-section or C-terminus of the proteins encoded by *B602L, B646L, F317L* and *MGF505-5R*. Data points indicate the number of spots per million cells induced by the pool of peptides after background subtraction, error bars indicate standard deviation from the mean. Note that to aid clarity the scale for the virus-specific responses (**A**) have been set to half the range of the peptide pools (**B**–**O**). Timing of boost (Bo) and challenge (Ch) are indicated on panels B to O.

**Figure 3:**
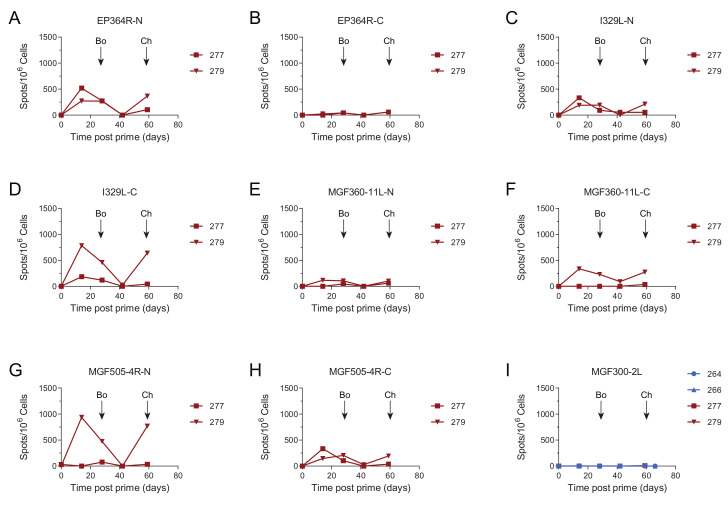
Antigen-specific IFNγ responses to Antigen Pool A (**I**, blue) or Antigen Pool B (**A**–**I**, red). Numbers of IFNγ-producing PBMC stimulated by pools of peptides were enumerated by ELIspot on the indicated days post-prime. N, mid and C indicate pools of peptides that correspond to the N-terminus, mid-section or C-terminus of the proteins encoded by *EP364R, I329L, MGF360-11L* and *MGF505-4R*. The response to *MGF300-2L*, an ASFV gene that was not part of either pool is also shown. Data points indicate the number of spots per million cells induced by the pool of peptides after background subtraction. Timing of boost (Bo) and challenge (Ch) are indicated.

Antigen Pool A induced ASFV-specific interferon gamma (IFNγ) secreting cells in the circulation, with pigs 264 and 266 having consistently higher numbers during the course of the experiment (Figure 2A). The levels of IFNγ secreting cells induced by Antigen Pool B were generally lower than those seen in animals immunized with Pool A, although responses above background were seen in all the animals except for 277. Consistent with our previous data, MVA boost did not significantly increase the numbers of ASFV-specific IFNγ secreting cells [21]. Pools of overlapping peptides corresponding to the individual proteins, or portions of the larger proteins, were used to identify antigen-specific cellular responses over time in two animals from each of the groups that were immunized with Antigen Pool A or Antigen Pool B (Figure 2 and Figure 3). Specific cellular responses were observed in at least one pig to all of the antigens in both Antigen Pool A and Antigen Pool B. 

### 3.2. Comparative Evaluation of Temperatures, Clinical Signs and Viremia Levels in Experiment 1

**Figure 4:**
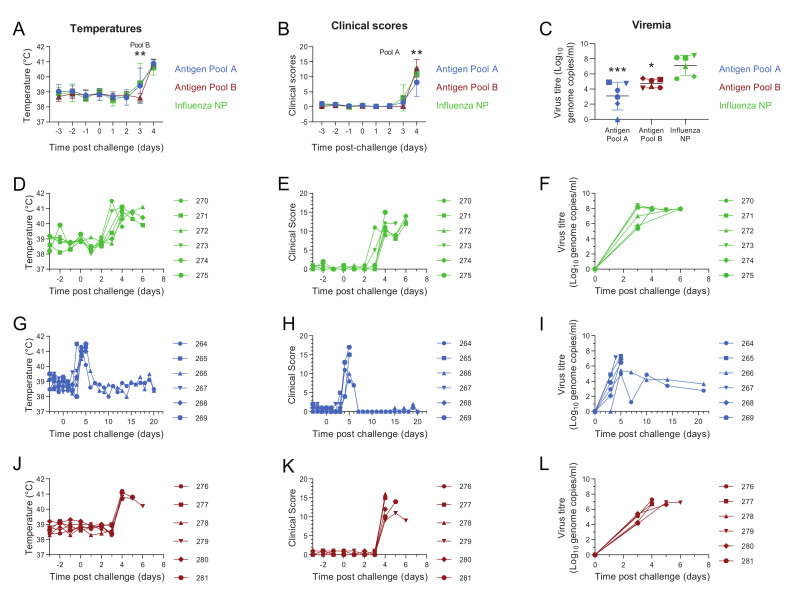
Clinical and virological parameters in experiment 1. Mean temperatures (**A**), clinical scores (**B**) up to four days and viremia three days post-challenge (**C**) of groups of six pigs primed and boosted with Antigen Pool A (blue), Antigen Pool B (red) or influenza NP (green). Temperatures (**D**,**G**,**J**) and clinical scores (**E**,**H**,**K**) for each individual pig immunized with influenza NP (**D**,**E**), Antigen Pool A (**G**,**H**) or Antigen Pool B (**J**,**K**) are shown. Blood was taken from animals immunized with Influenza NP (**F**), Antigen Pool A (**I**), Antigen Pool C (**L**) on the indicated days and DNA was extracted in duplicate and the viral titer determined by qPCR, with each extraction tested in duplicate. Data points are the mean of the duplicate extractions. Errors bars on panels A, B and C indicate standard deviation from the mean. Asterisks on panels B and C show significant differences in the indicated group of pigs compared to the control. Temperatures and clinical scores were assessed by repeated-measures two-way ANOVA, * *p* ≤ 0.05, ** *p* ≤ 0.01 or *** *p* ≤ 0.001.

Beginning three days post-challenge with the virulent OUR T88/1 strain of ASFV, pigs immunized with Antigen Pool A or influenza NP showed body temperatures above 40.5 °C (Figure 4D,G). Four days post-challenge temperatures above 40.5 °C, and in many cases above 41 °C, were detected in the rest of pigs belonging to the different experimental groups (Figure 4D,G,J). Increases in temperature were accompanied by an increase in non-specific clinical signs (lethargy, reduced appetite) whose severity increased as the disease progressed. With the exception of pigs 264 and 266 immunized with Antigen Pool A, which showed a decrease in temperatures and clinical scores from six days post-challenge (Figure 4G,H), temperatures and clinical scores remained high in the rest of pigs belonging to the different experimental groups, so that animals were euthanized after reaching the humane end-point between four and six days post-challenge. However, pigs 264 and 266 immunized with Antigen Pool A were maintained for twenty days after challenge and did not show any clinical signs after they had recovered from fever during the first week.

Average temperatures (*p* = 0.0025; F = (7, 105) = 39.94) and clinical scores (*p* = 0.0085; F (7, 105) = 90.05) in animals immunized with Antigen Pool B were lower than the controls three days post-challenge, but not on the following day (Figure 4A,B). The clinical scores observed in the controls were higher than those in animals immunized with Antigen Pool A (*p* = 0.0085; F (7, 105) = 90.05) four days post-challenge. Three days post-challenge, viremia levels were approximately 10,000 or 1000 less than the controls in the groups of pigs immunized with Antigen Pool A and Antigen Pool B respectively (one-way ANOVA, F (2, 15) = 15.32; *p* = 0.0003 and 0.0208 respectively). Viremia peaked five days post-challenge in pigs 264 and 266 that survived until the end of the experiment and were lower than those detected in the pigs that were euthanized. From six days post-challenge, a progressive decrease in viremia levels was observed in these two animals. Despite the absence of clinical signs and temperatures over this period of time, low levels of infectious virus persisted in pigs 264 and 266 until the end of the experiment (Figure 4I and Figure S4). 

### 3.3. Evaluation of Macroscopic Lesions and Viral Load in the Tissues in Experiment 1

During necropsies, a descriptive evaluation of lesions was carried out, but without following any scoring system. Apart from protected pigs given Pool A (pigs 264 and 266), the rest of the pigs immunized with Pool A (4 of 6 pigs) and Pool B (6 of 6 pigs) showed macroscopic lesions characteristic of acute forms of ASF such as mild hydropericardium and pulmonary congestion, mild to moderate ascites, mild to moderate hyperemic splenomegaly, lymphadenopathy with petechiae as well as hemorrhagic lymphadenopathy that affected mainly renal and gastrohepatic lymph nodes, and renal hemorrhages among others lesions. Significant differences were found in the viral load in some tissues of animals immunized with the different pools of antigens and those seen in the controls (Figure S5). The viral yield was approximately 1000-fold lower in the gastro-hepatic lymph node of the animals given Antigen Pool A and 100-fold lower than animals given Pool B compared to the controls. Compared to the controls viral load was greater than 100-fold less in the retropharyngeal lymph node of pigs immunized with either Antigen Pool A or Pool B. These differences were still significant if the two pigs that survived (264 and 266) and were culled two weeks later than the other animals, were omitted from the analysis.

### 3.4. Immune Responses after Challenge in Surviving Animals in Experiment 1

Titers of ASFV-specific antibodies measured by IFA did not increase seven days post-challenge (66 days post-prime) in comparison to their pre-challenge levels (Figure 1A). This was also reflected in the responses to the individual proteins that were measured by indirect ELISA, *B602L*, *CP204L* and *E183L* (Figure 1C,E,F). No increase in IgG specific for *B602L* and *CP204L* after the challenge was detected in the sera of pigs 264 and 266 (Figure S6). This suggested that challenge with virulent ASFV did not provoke a secondary humoral immune response, although we did not test the response to all of the proteins that comprised Antigen Pool A. There was no response to *B646L* prior to challenge, however high ODs were observed in ELISAs at the end of the study to *B646L* and also to *B602L* and *CP204L*. Pigs that have recovered from natural infection with ASFV typically have *B646L*-specific antibodies in the circulation [45] and the absence of an increase in the response to any of the proteins we tested 7 days post-challenge makes it possible that the antigen-specific responses detected at the end of the experiment represent primary immune responses to the virus, rather than secondary responses to the individual antigens. Numbers of ASFV-specific IFNγ secreting cells in the peripheral blood increased after challenge in both animals 264 and 266 (Figure 2A, F (3, 12) = 33.23; *p* = 0.0061 and *p* = 0.0054 respectively). However, the response to the individual antigens appeared to be dependent on the pig with 266 showing increased responses to pools of peptides corresponding to *CP204L* (Figure 2B), B646L (Figure 2H) and *EP153R* (Figure 2K) seven days post-challenge (day 66). Analysis of the responses in pig 264 was limited due to the lack of viable cells pre-challenge, however, unlike animal 266 only the responses to *B602L* and *EP153R* out of the individual antigens were higher on day 66 than they were two weeks post-boost (day 42). 

### 3.5. Immune Responses in Experiment 2

Both Antigen Pool A and Pool B induced virus- and antigen-specific immune responses (Figure 1, Figure 2 and Figure 3) and both groups of animals showed reduced clinical signs and viremia or viral load in the tissues relative to the controls (Figure 4A–C). Clearly, the most striking difference was seen in the two animals vaccinated with Pool A (264 and 266) which recovered from a transient fever and were then clinically normal until the end of the experiment. Pigs 264 and 266 had elevated ASFV-specific IFNγ secreting cells in the bloodstream two weeks post-boost relative to the other animals immunized with Antigen Pool A (Figure 2A) therefore we considered that increasing the dose of viral vectors or reducing the number of antigens in the pool may lead to an increase in the cellular response after prime and boost. In experiment 2, groups of six pigs were immunized with Antigen Pool A using the same schedule as in experiment 1 except the dose of each rAd was increased three-fold to 1.5 × 10^10^ IU and each MVA two and half times to 2 × 10^8^ pfu per dose. Antigen Pool C comprised a sub-pool of Antigen Pool A and included *B646L, CP204L, E199L, F317L* and *MGF505-5R*.

**Figure 5:**
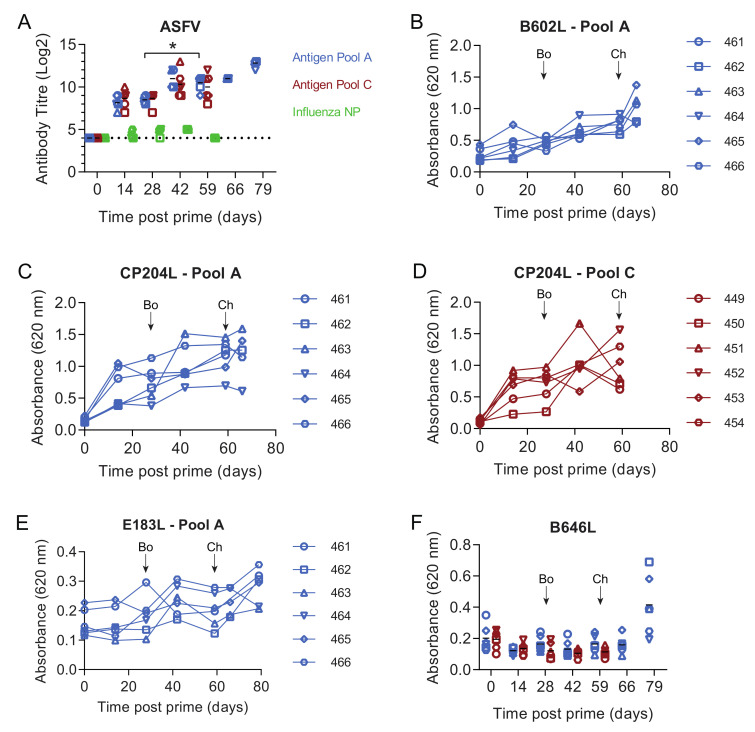
Antibody responses in pigs from experiment 2 primed with rAd encoding Antigen Pool A (blue), Antigen Pool C (red) or influenza NP. Animals were boosted with rAd/MVA 28 days after priming and challenged with ASFV 59 days after priming. (**A**) The titre of anti-ASFV antibodies in the serum of the indicated animals was determined by immunoperoxidase assay. Antibodies to ASFV proteins *B602L* (**B**), *CP204L* (**C**,**D**), *E183L* (**E**) and *B646L* (**F**) were detected in 1:100 dilutions of serum collected from the indicated animals at the indicated times post-prime by ELISA, and expressed as the absorbency at 620 nm. Timing of boost (Bo) and challenge (Ch) are indicated on panels B to F. Data points on panel F represent values from individual animals and are labelled as on panels D (Antigen Pool C) and E (Antigen Pool A). Differences between antibody titres were assessed by repeated-measures two-way ANOVA,* *p* ≤ 0.05.

As seen in experiment 1, ASFV-specific antibodies were observed by IFA in the groups of pigs immunized with the two antigen pools after prime and these were elevated after the boost (Figure 5A). Titers were higher on day 59 when compared to day 28 in pigs immunized with Antigen Pool A, but not in those immunized with Antigen Pool C (F (2.467, 24.67) = 25.13; *p* = 0.0239 and *p* = 0.1407 respectively) suggesting that the boost had a small effect on the levels of ASFV-specific serum antibodies. There was no difference in the titers between the two groups of pigs, nor when titers were compared to those seen in experiment 1, suggesting the increased dose of virus vectors in the prime and boost did not affect the levels of ASFV-specific antibodies. Antibodies specific for B602L and *CP204L* were detected and the response to *E183L* and *B646L* was poor (Figure 5B–F), antibodies to *F317L* were also detected by IFA, but antibodies to *EP153R*, *E199L* or *MGF505-5R* were not (not shown). Although there was no difference in the titers of ASFV-specific antibodies between experiments 1 and 2, significant differences were seen between the response to B602L and CP204L (Figure S7) with higher ODs observed in experiment 2.

**Figure 6:**
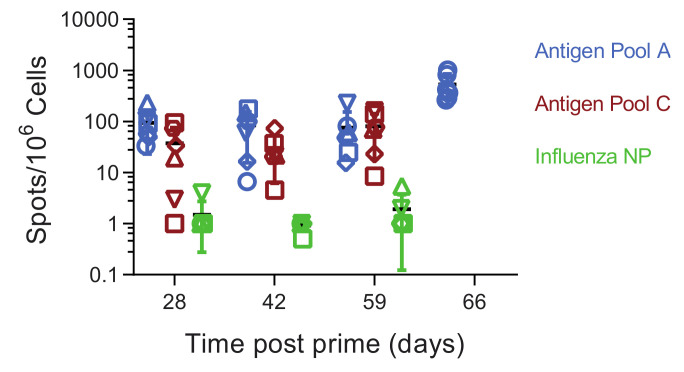
Virus-specific IFNγ responses in animals from experiment 2 immunized with Antigen Pool A (blue), Antigen Pool B (red) or influenza NP (green). The number of ASFV-specific IFNγ producing PBMC were enumerated following in vitro stimulation with ASFV by ELIspot on the indicated days post-prime. Data points indicate the number of spots per million cells induced by the whole virus after background subtraction, error bars indicate standard deviation from the mean. Note that to aid clarity the scale for the virus-specific responses have been set differently to those in Figure 2.

Analysis of the cellular response showed that immunization primed ASFV-specific IFNγ-secreting cells in both groups of pigs (Figure 6). The boost did not increase the numbers of IFNγ-secreting cells and there were no differences between the numbers of ASFV-specific, IFNγ-secreting cells in the pigs immunized with Antigen Pool A and those given Antigen Pool C. Comparing the responses induced by Antigen Pool A between experiment 1 and 2 showed that increasing the dose did not increase the number of circulating ASFV-specific, IFNγ-secreting cells. Antigen-specific cellular responses were detected to all of the individual antigens in the pigs that were tested (Figure S8), however, the numbers of *EP153R* specific responses were very low in all of the animals except in pig 463, which also had a poor response to *B602L*. There were no qualitative differences in the responses to individual antigens between the pigs that we were able to test that had been immunized with either Antigen Pool A or Antigen Pool C, although unfortunately there was insufficient material to test all of the animals and perform a thorough statistical analysis. Taken together, Antigen Pool A and Antigen Pool C delivered by rAd and boosted with a combination of rAd and MVA induced ASFV-specific cellular immune responses that were indistinguishable from each other. Antigen-specific responses two weeks after boost (day 42) determined in an additional two pigs from experiment 1 were used to make a comparison of cellular responses between the two experiments (Figure S7B). No differences were observed with the exception of *MGF505-5R* where the number of IFNγ secreting cells was slightly higher in experiment 1.

### 3.6. Antigen Pool A Protected 100% of Pigs from Fatal Disease

**Figure 7:**
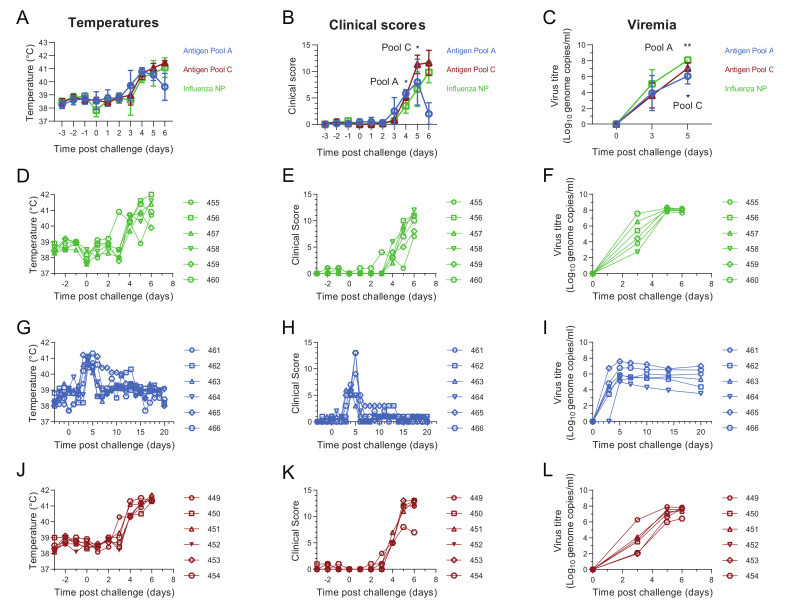
Clinical and virological parameters in experiment 2. Mean temperatures (**A**), clinical scores (**B**) up to six days and viremia up to five days post-challenge (**C**) of groups of six pigs primed and boosted with Antigen Pool A (blue), Antigen Pool C (red) or influenza NP (green). Temperatures (**D**,**G**,**J**) and clinical scores (**E**,**H**,**K**) for each individual pig immunized with influenza NP (**D**,**E**), Antigen Pool A (**G**,**H**) or Antigen Pool C (**J**,**K**) are shown. Blood was taken from animals immunized with Influenza NP (**F**), Antigen Pool A (**I**), Antigen Pool C (**L**) on the indicated days and DNA was extracted in duplicate and the viral titer determined by qPCR, with each extraction tested in duplicate. Data points are the mean of the duplicate extractions. Errors bars on panels A, B and C indicate standard deviation from the mean. Black asterisks on panels B and C show significant differences (repeated measures two-way ANOVA) of the indicated group of pigs compared to the control, * *p* ≤ 0.05 or ** *p* ≤ 0.01. Red asterisks on panels A and B show significant differences (repeated measures two-way ANOVA) between pigs immunized with Antigen Pools A and B six days post-infection, * *p* ≤ 0.05 or **** *p* ≤ 0.0001.

Four weeks after boost, all animals were challenged with the virulent OUR T88/1 strain of ASFV. Three days after the challenge, pigs in all experimental groups developed pyrexia, with temperatures in some cases above 40.5 °C (Figure 7D,G,J). Temperatures increased progressively, surpassing 40.5 °C between four and five days post-challenge in most of the animals and reaching in many cases values above 41 °C. The increase in temperature of the animals was accompanied by an increase in clinical signs of disease ranging from non-specific (lethargic animals, reduced appetite) to more severe clinical signs as the disease progressed (animals not eating and reluctant to get up or move, increased breathing rate, blood in feces, vomiting). Clinical scores were higher in pigs immunized with Antigen Pool A when compared to those in the control group four days post-challenge (*p* = 0.0170; F (18,135) = 10.49) whereas clinical scores in pigs immunized with Antigen Pool C were higher than those seen in pigs in the control group (*p* = 0.0376; F (18, 135) = 10.49) five days post-challenge (Figure 7B). 

In experiment 1 animals immunized with either Antigen Pool A or Antigen Pool B had significantly reduced viremia 3 days post-challenge compared to the controls (Figure 4C). This coupled with the apparent enhanced clinical signs seen in the two vaccinated groups in experiment 2 led us to speculate that the observed disease progression could be due to an overreaction of the immune system. Blood samples from the animals in experiment 2 that were immunized with the two antigen pools were tested for the presence of ASFV antigen five days post-challenge using lateral flow devices. Only animals immunized with Antigen Pool C were positive, with pig 449 clearly positive and pigs 450, 451 and 452 weakly positive. The other two animals immunized with Antigen Pool B and all of the animals immunized with Antigen Pool A were negative. Temperatures had risen between day 4 and day 5 in all of the animals immunized with Antigen Pool B and two of the animals immunized with Antigen Pool A, therefore, in order to mitigate clinical signs, a single treatment with a nonsteroidal anti-inflammatory and antipyretic drug flunixin meglumine (Finadyne, 2 mL per 45 kg bodyweight) was administered on day 5 post-challenge to all pigs that had been immunized with either Antigen Pool A or C. The controls were not treated as previous experience with the OUR T88/1 strain has shown that animals can die suddenly as early as five days post-challenge [32] and therefore any potential masking of clinical signs that could lead to the death of an animal would have been ethically unacceptable. The treatment did not mitigate clinical signs in pigs immunized with Antigen Pool C so these animals were euthanized, along with the pigs in the control group, the following day (6 days post-challenge). However, temperatures and clinical signs progressively decreased in all pigs immunized with Antigen Pool A reaching normal values, which were maintained until the animals were euthanized twenty days after challenge.

ASFV genome was detected in all pigs after challenge (Figure 7F,I,L), viremia generally increased as temperatures and clinical signs progressed, peaking at five or six days post-challenge. Viremia in the group of pigs immunized with Antigen Pool A or Pool C (F (4, 30) = 2.042; *p* = 0.0080 and *p* = 0.0330 respectively) were significantly reduced compared to the controls at five days post-challenge (Figure 7C). From seven days post-challenge, a progressive decrease in viremia levels was observed in the pigs immunized with Antigen Pool A. Animal 465 that maintained the highest viremia until the end of the experiment also maintained a temperature of 40 °C up to 11 days post-challenge. Infectious virus persisted in all the animals immunized with Antigen Pool A until the end of the experiment (Figure S4B). 

### 3.7. Evaluation of Macroscopic Lesions in Experiment 2

Macroscopic lesions were assessed during necropsies following scoring methods based on previous standardized protocols [43]. Most of the pigs included in the control group and those vaccinated with Antigen Pool C, euthanized at 6 days post-challenge, displayed macroscopic lesions characteristic of acute forms of ASF such as mild to moderate hyperemic splenomegaly (12/12), hemorrhagic lymphadenitis (11/12) that affected mainly renal (11/12) and gastrohepatic lymph nodes (5/12) or skin erythema and cyanosis on the skin of the ears (5/12) (Figure S9). On the other hand, pigs immunized with Antigen Pool A, which survived until the end of the experiment, only showed nonspecific mild macroscopic changes that were not characteristic of ASF.

### 3.8. Antibody and Cellular Responses after Challenge in Pigs Immunised with Antigen Pool A in Experiment 2

The number of circulating ASFV-specific IFNγ-secreting cells increased between day 59 (pre-challenge) and day 66 (seven days post-challenge) in all of the pigs immunized with Antigen Pool A except for animal 464, however, taken as a group there were no significant difference between the two-time points (*p* = 0.0721, repeated measures one-way ANOVA; F (1.226, 6.13) = 10.50). The response to each antigen varied between each pig with, for example, animal 463 showing an increase to all of the antigens, whereas 461 only showed an increase to *CP204L* and *B602L* (Figure S8). No increase in the response to any of the antigens was detected in cells from animals 465 and 466, despite an increase in the response to the whole virus. This may suggest that the increase in the number of ASFV-specific IFNγ secreting cells between day 59 and 66 is, in fact, a primary response to viral infection, rather than a secondary response to the immunization regime. In addition, there was no significant increase in the total antibody response to *B602L* (Figure 5B) or CP204L (Figure 5C) or IgG specific to the two proteins between day 59 and day 66 (Figure S6). As seen in experiment 1, most of the surviving pigs had antibodies specific for *B646L*/p72 at the end of the experiment despite having none prior to challenge (Figure 5E). Taken together there was no clear correlate of protection in the immune responses that were tested.

### 3.9. Clinical Outcomes Did Not Correlate with Inferred SLA Genotypes

The lack of clear correlates of protection led us to consider the genetic background of the animals used in the two studies. Differential protection induced by a live attenuated ASFV has been observed between *NIH* cc and dd minipigs that are inbred for different swine leukocyte antigen (*SLA*) class I and class II molecules [26]. In addition, the eight antigens were originally selected for their immunogenicity in the inbred Large White Babraham line of pigs which are homozygous at the *SLA* locus [29,46]. Low resolution genotyping of the twenty-four pigs immunized with Antigen Pools A, B and C identified ten *SLA* class I and eleven *SLA* class II haplotypes (Table 3). Two *SLA* class I and one *SLA* class II haplotypes were not resolved. *SLA* class I haplotype Lr-22.0 occurred in eight of the twenty-four pigs with Lr-4.0 and Lr-24.0 found in seven and five pigs respectively. Lr-0.15b was the most common *SLA* class II haplotype, being identified in eleven animals, followed by Lr-0.14 and Lr-0.19a which were found in seven and six pigs respectively. There was no clear correlation between the observed clinical outcomes and the *SLA* genotype of the respective animals. Pigs 265 and 266 that were immunized with Antigen Pool A in experiment 1 had similar *SLA* genotypes, however, 266 survived to the end of the experiment whereas 265 was euthanized five days post-challenge. The animals immunized with Antigen Pool A in experiment 2 that survived until the end of the experiment expressed a range of *SLA* alleles. Pigs 461 and 465 were homozygous for class II haplotype Lr-0.15b and 463 was homozygous for class I haplotype Lr-4.0. Interestingly, 461 and 465 that were immunized with Antigen Pool A and 449 that was immunized with Antigen Pool C had the same inferred *SLA* haplotype suggesting that the *SLA* genotype did not correlate with the observed clinical outcome between the two different pools of antigens. Pig 278 that was immunized with Antigen Pool B in experiment 1 was not protected despite encoding the same *SLA* haplotype as the Babraham line (Lr-55.6). Taken together, the results suggested that the different pools of antigens identified in an inbred pig line were immunogenic across a range of *SLA* genotypes and, although the number of animals used was low, that the *SLA* genotype did not correlate with clinical outcomes after the challenge. 

## 4. Discussion

Live attenuated virus vaccines induce robust immunity against related strains of ASFV, but have suffered from a poor safety profile in the past [47]. More recent live attenuated strains generated by targeted gene deletion do not induce chronic forms of the disease, but a reversion to virulence is still a concern [10]. A subunit vaccine against ASFV would avoid these potential complications, be able to differentiate infected from vaccinated animals (DIVA), and depending on the vaccine platform is likely to be easier to manufacture than fully replicating ASFV. Recent work to identify immunogenic proteins encoded by ASFV has significantly increased the range of potential proteins that could be included in a subunit vaccine. However, the absence of reliable correlates of protection or a thorough understanding of the mechanisms of immunity force a reliance on an essentially empirical approach to selecting protective antigens. Here, we present the results of two immunization and challenge experiments using pools of viral vectored ASFV genes that ultimately led to 100% protection against a fatal disease. 

Twelve antigens were selected based on their ability to induce immune responses in inbred Babraham pigs using a DNA-prime VACV-boost regime [29]. We have previously shown that viral vectored *B646L* and *CP204L* are immunogenic in outbred pigs [21] and others have shown rAd expressing *B602L* and *E183L* induced humoral and cellular immune responses in domestic swine [18,19,22]. Despite observing a cellular response, we were unable to detect antibody responses to *B646L* in rAd-primed, MVA-boosted pigs despite other studies demonstrating *B646L*-specific antibody responses after homologous rAd prime and boost [18,19,22]. The antibody response we detected to *E183L* was also poor compared to that seen by other researchers. One explanation for these discrepancies is that we did not use adjuvant in our experiments. Another possibility is that the combination of homologous rAd prime and boost was more effective at inducing antibody responses against *B646L* and *E183L* than heterologous rAd prime and MVA boost used in this study. MVA-vectored *EP153R* induced cellular responses in the absence of antibody response [20], but to our knowledge, the remaining antigens have not been tested in domestic swine and we were able to detect cellular immune responses against *E199L*, *EP364R*, *F317L*, *I329L*, *MGF360-11L*, *MGF505-4R* and *MGF505-5R*. We have now tested the immunogenicity of twenty-eight different ASFV genes using viral vectors and have shown that in the majority of cases they induce antigen-specific cellular responses in pool sizes of up to twelve vectors [21]. Analysis of the inferred *SLA* haplotypes showed that results from immunogenicity studies with inbred pig lines can be applied effectively to outbred domestic swine.

The different combinations of antigens and doses tested in these experiments all induced cellular and humoral immune responses that were capable of recognizing the whole virus. However, no clear correlate of protection emerged that could predict the clinical outcomes after challenge with virulent ASFV. The two animals that were protected against fatal disease in experiment 1 had the highest number of circulating ASFV-specific, IFNγ-secreting memory cells, however, pigs in experiment 2 immunized with Antigen Pool A that were protected had similar numbers of ASFV-specific, IFNγ-secreting memory cells to those that were not protected in experiment 1. In addition, animals immunized with Antigen Pool C in experiment 2 had similar levels of ASFV-specific, IFNγ-secreting cells and these were not protected either. The limited protection that we observed with other pools of viral vectored antigens [21] were only observed in animals with significant numbers of PBMC that secreted IFNγ after in vitro stimulation with the whole virus. Therefore, it is tempting to speculate that priming of ASFV-specific, IFNγ-secreting cells is necessary, but not sufficient to mediate protective immunity. Further experiments to phenotype the cells that secrete IFNγ, to determine if they also secrete interleukin-2 and tumour necrosis factor-α simultaneously after activation, and to determine if they exhibit ASFV- or antigen-specific cytotoxicity, may refine our understanding of the importance of the cellular response in protecting animals against severe disease.

We were unable to correlate ASFV-specific or antigen-specific antibody responses in individual pigs to protection, although we did not examine the antibody responses to all of the antigens in detail and did not determine end-point titers for the antigens in the different pools. We were also unable to show any effect of serum from any of the animals in these experiments on the ability of OUR T88/1 to infect macrophages (not shown) [48]. However, it is important to note that we have no positive control for these neutralization experiments and therefore it is possible that neutralizing or infection-enhancing antibodies were induced in these animals and could have played a role in the observed clinical outcomes. Conformational neutralizing antibodies against *B646*L have been described [49] and generating such an immune response will likely require the formation of the trimers in which the protein is found in assembled virions [50,51,52]. Formation of *B646L* trimers is dependent on co-expression of *B602L* [53,54], however, ASFV neutralizing antibodies targeting *E183L* and *CP204L* can be induced in pigs by recombinant protein alone [13]. Further experiments should also test for antibody-dependent cellular cytotoxicity as this functionality has been observed in animals that have naturally recovered from ASF [55]. The observation that the group of pigs immunized with Antigen Pool A in experiment 2 had a more robust anti*-CP204L* and *-B602L* antibody response when compared to experiment 1 suggests further studies on the importance of the antibody response are warranted.

Differences in the number of protected animals immunized with Antigen Pool A between experiments 1 and 2 could have either been due to the increased dose of vectors used, the treatment with flunixin meglumine five days post-challenge in experiment 2, or the genetic background of the animals themselves. No evidence in support of the latter point was found after *SLA* haplotyping, but many other factors play a role in the response to immunization and challenge. The temperatures of pigs 463, 464 and 465 in Antigen Pool A had already begun to decrease from their maximum before treatment with flunixin meglumine, pig 462 maintained a temperature of 40.4 °C before and after the treatment and the temperature in pig 465 increased after intervention with flunixin meglumine. Pigs 461 and 466 had high temperatures and had shown disinterest in food the day before treatment and both showed improvement afterwards. Therefore, it is possible that the flunixin meglumine may have contributed to these animals not reaching their humane end-point six days post- challenge. However, treatment with flunixin meglumine did not prevent temperatures increasing in the animals immunized with Antigen Pool B and all animals in this group that refused food prior to treatment refused the day after. This is consistent with data from others that show continual treatment with flunixin meglumine does not alleviate clinical signs induced by virulent ASFV [56]. Therefore, treatment of the animals immunized with Antigen Pool A in experiment 2 may have prevented some of the pigs reaching their humane endpoint six days post-challenge, however, it is unlikely to have influenced their ultimate survival. One potential difference was that some antigen-specific antibody responses were higher in experiment 2 than in experiment 1 suggesting the increased dose of the vector may have improved individual responses without leading to an increase in the titer of ASFV-specific antibodies. More detailed analysis of the responses to the other proteins in Antigen Pool A, in conjunction with improved tools to study the function of antibodies to the whole virus will help us to determine if the enhanced responses observed to a couple of antigens are representative of a wider importance of the antibody response in protection.

One of the key questions is which of *B602L, B646L, CP204L, E183L, E199L, EP153R, F317L* and *MGF505-5R* in Antigen Pool A are required for protection and as *B*6*02L, E183L* and *EP153R* were absent from Antigen Pool C it is likely that one or more of these play an important role. *B602L* and *EP153R* were present in Antigen Pool B and this did not confer any protection from severe disease, although viremia was slightly reduced compared to the control group in this experiment. However, it is possible that some of the other antigens in Pool B may have interfered with any protective immune responses induced by expression of *B602L* and *EP153R*, as we and others have seen vaccine-induced disease enhancement with viral vectors [21,22]. In other studies, *E183L* was part of the fusion protein used in DNA vaccination studies that induced partial protection [15] and was included in a pool of rAd-vectored ASFV genes along with *B646L* and *CP204L* that may protect pigs [22]. Therefore, it is possible that *E183L* may be an important protective antigen that was absent from Antigen Pool C. *B646L* and *CP204L* were included with another ten antigens in a previous viral vectored vaccine study, but did not protect animals from reaching the humane endpoints of the study [21]. *F317L* and *MGF505-5R* were present in all three pools of antigens tested in this study which may argue for a less important role in protection. It is likely that antigens within Antigen Pool C in combination with one or more of *B602L* and *E183L*, *EP153R* mediate the protective immune response observed in these experiments. All of the surviving animals showed clinical signs after challenge and all of them were viremic at the end of study, future experiments should determine whether pigs are able to clear the infection and also if these vaccinated animals shed infectious virus that could be transmitted to naïve animals. It is likely that additional antigens will need to be identified to provide an immune response capable of further suppressing viral replication. 

Genotype I viruses have proved useful model systems to study the protective immune response to ASFV [2,28,57] and the genes we have used in our studies are derived from the genotype I isolates OUR T88/3 and Benin 1997/1. Outbreaks of ASF in Eurasia since 2007 have been caused by a genotype II virus, and cross-protection between ASFV isolates is difficult to predict [27,28,32]. With the exception of *B602L* and *EP153R*, the identity between the Georgia 2007/1 protein sequences and those used in this study are greater than 90% and the low virulent NH/P68 genotype I isolate can protect against challenge with virulent genotype II virus [58,59] suggesting genotype I antigens may protect against genotype II challenge, although there may be differences in critical T-cell or antibody epitopes. Genotype I viruses continue to cause outbreaks across western sub-Saharan Africa [60,61,62,63,64] and an effective vaccine would provide an additional control measure in this region that could aid commercial and subsistence farmers from the devastation caused by ASF [62,64]. In order to realize such a vaccine, future work will need to concentrate on refining the specific antigens within the 8 identified that are required for protection against fatal disease, defining correlates of protection, determining the requirement for a boost, as well as identifying other antigens to improve the efficacy of the immunization regime.

## 5. Conclusions

Viral vectors represent an attractive vaccine platform due to their ability to induce robust antigen specific humoral and cellular immune responses as well as their excellent safety record. Adenovirus, alphavirus and vaccinia virus vectors have all been shown to induce effective ASFV-antigen specific responses [18,19,20,21,22,23,65]. However, despite the promising immunogenicity data these vaccination regimes have shown limited protection after challenge with virulent ASFV and in some cases resulted in enhanced disease. We have identified eight ASFV genes that when delivered to pigs using an adenovirus prime and modified vaccinia Ankara boost can protect pigs against a fatal disease caused by a genotype I ASFV strain. This data may provide the basis for development of an effective subunit vaccine against African swine fever.

## Figures and Tables

**Table 1:** Composition of Antigen Pools. The individual HA-tagged ASFV genes in each pool and the dose of each individual vector used in the prime and/or boost are indicated. Genes highlighted in bold were administered by rAd prime and MVA boost whereas those shown in italics by rAd prime and rAd boost.

Experiment	1		2	
Antigen Pool	A	B	A	C
Genes	*B602L*	*B602L*	*B602L*	*B646L*
	*B646L*	*EP153R*	*B646L*	*CP204L*
	*CP204L*	*EP364R*	*CP204L*	*E199L*
	*E183L*	*F317L*	*E183L*	*F317L*
	*E199L*	*I329L*	*E199L*	*MGF505-5R*
	*EP153R*	*MGF360-11L*	*EP153R*	
	*F317L*	*MGF505-4R*	*F317L*	
	*MGF505-5R*	*MGF505* *-5R*	*MGF505-5R*	
rAd Dose (IU)	5 × 10^9^	5 × 10^9^	1.5 × 10^10^	1.5 × 10^10^
MVA Dose (pfu)	7.5 × 10^7^	7.5 × 10^7^	2 × 10^8^	2 × 10^8^

**Table 2:** Vero cells transfected with the individual antigens were stained with serum taken from the individual pigs before immunization or before the challenge. Wells were incubated with serum diluted 1:100 and anti-V5 antibody, then goat anti-pig and -mouse antibodies conjugated to Alexa Fluor 488 and 568 respectively. A pig was considered positive for a given antigen if the pre-immunization sera were negative and the pre-challenge sera were positive. A dash indicates the sample was not tested against this protein.

Protein	Antigen Pool A						Antigen Pool B					
	264	265	266	267	268	269	276	277	278	279	280	281
*E199L*	No	No	No	No	No	No	-	-	-	-	-	-
*EP153R*	No	No	No	No	No	No	No	No	No	No	No	No
*EP364R*	-	-	-	-	-	-	No	No	No	No	No	Yes
*F317L*	Yes	Yes	Yes	Yes	Yes	Yes	Yes	Yes	Yes	Yes	Yes	Yes
*I329L*	-	-	-	-	-	-	Yes	Yes	Yes	No	No	Yes
*MGF360-11L*	-	-	-	-	-	-	No	No	No	No	No	No
*MGF505-4R*	-	-	-	-	-	-	No	No	No	No	No	No
*MGF505-5R*	No	No	No	No	No	No	No	No	No	No	No	No

**Table 3:** *SLA* genotypes and haplotypes of pigs. Animals 264 through 269 from experiment 1 and 461 through 466 from experiment 2 were immunized with Antigen Pool A, animals 276 through 281 from experiment 1 with Antigen Pool B and animals 449 through 454 from experiment 2 were immunized with Antigen Pool C. *SLA* genotypes of three class I genes; *SLA-1*, *SLA-2* and *SLA-3* and three class II genes; *DRB1*, *DQB1* and *DQA* were determined from each pig by PCR-SSP typing. *SLA* genotypes are shown as low-resolution allele groups (e.g., *SLA*-1*04:XX), intermediate-resolution allele strings (e.g., DRB1*04:03–04) or specific high-resolution alleles (e.g., DQB1*02:01), for example, pig 266 bears the SLA-1*16:03 allele and one of the SLA-1*04:XX group alleles; up to two different alleles from the SLA-3*04:XX group; and one allele from the SLA-2*04:XX group and one from the SLA-2*06:XX. The inferred class I and class II SLA haplotype is also shown for each pig, with the high-resolution haplotype data for the Babraham (Bab) shown for reference. Pigs that recovered after challenge are highlighted by grey shading.

Exp. Group	Pig ID	SLA I Allele Specificity			SLA II Allele Specificity			Inferred Haplotype		
		SLA-1	SLA-3	SLA-2	DRB1	DQB1	DQA	Class I	Class II	Complete
Bab	Reference	14:02	04:03	11:04	05:01	08:01	01:03	55/55	6/6	55.6/55.6
Pool A	264	04:XX,07:XX	04:XX	02:XX,04:XX	06:01,09:XX	07:XX,08:XX	01:XX,03:XX	4/32	12b/14	4.14/32.12b
	265	04:XX,16:03	04:XX	04:XX,06:XX	02:XX,04:03-04	04:XX,07:XX	02:XX,03:XX	4/24	4/19a	4.4/24.19a
	266	04:XX,16:03	04:XX	04:XX,06:XX	01:XX,04:03-04	01:XX,07:XX	01:XX,03:XX	4/24	1/19a	4.1/24.19a
	267	08:XX,14:01	04:XX,06:01	06:XX,12:XX	04:XX,06:XX	02:02,07:XX	01:XX,02:XX	22/62	12a/15b	22.15b/62.12a
	268	04:XX,08:XX	04:XX,06:01	04:XX,12:XX	01:XX,04:XX	01:XX,02:02	01:XX,02:XX	4/22	1/15b	4.1/22.15b
	269	08:XX,09:XX,15:XX	06:01,07:XX	05:XX,12:XX	04:XX,10:XX	02:02,06:XX	01:XX,02:XX	22/28	15b/23	22.15b/28.23
Pool B	276	08:XX,16:03	04:XX,06:01	06:XX,12:XX	04:XX,04:03-04	02:02,07:XX	02:XX,03:XX	22/24	15b/19a	22.15b/24.19a
	277	Blank/undetected	05:XX	06:XX	09:XX,11:XX	04:XX,08:XX	02:XX,03:XX	33/33	14/26	33.14/33.26
	278	04:XX,14:02	04:XX	04:XX,11:04-05	01:XX,05:XX	01:XX,08:XX	01:XX	4/55	1/6	4.1/55.6
	279	11:XX,12:XX,13:01	04:XX,05:XX	04:XX,10:XX	04:03-04,09:XX	03:02-03,08:XX	02:XX,03:XX	35/43	13/14	35.13/43.14
	280	11:XX,12:XX,13:01	04:XX,05:XX	04:XX,10:XX	04:03-04,09:XX	03:02-03,08:XX	02:XX,03:XX	35/43	13/14	35.13/43.14
	281	08:XX,09:XX,15:XX	06:01,07:XX	05:XX,12:XX	04:XX,10:XX	02:02,06:XX	01:XX,02:XX	22/28	15b/23	22.15b/28.23
Pool A	461	07:03,08:XX	06:01	05:XX,12:XX	04:XX	02:02	02:XX	21/22	15b/15b	21.15b/22.15b
	462	07:03,11:XX	04:XX,06:01	04:XX,05:XX	04:XX,09:XX	02:02,08:XX	02:XX,03:XX	21/43	14/15b	21.15b/43.14
	463	04:XX	04:XX	04:XX	02:XX,09:XX	04:XX,08:XX	02:XX,03:XX	4/4	4/14	4.4/4.14
	464	Blank/undetected	05:XX,07:XX	05:XX,06:XX	10:XX,11:XX	06:XX	01:XX	*/33	23/26	* 23/33.26
	465	07:03,08:XX	06:01	05:XX,12:XX	04:XX	02:02	02:XX	21/22	15b/15b	21.15b/22.15b
	466	12:XX,13:01,16:03	04:XX,05:XX	06:XX,10:XX	04:03-04,10:XX	06:XX,07:XX	01:XX,03:XX	24/35	19a/23	24.19a/35.23
Pool C	449	07:03,08:XX	06:01	05:XX,12:XX	04:XX	02:02	02:XX	21/22	15b/15b	21.15b/22.15b
	450	11:XX,14:01	04:XX	04:XX,06:XX	06:XX,09:XX	07:XX,08:XX	01:XX,03:XX	43/62	12a/14	43.14/62.12a
	451	04:XX,07:03	04:XX,06:XX	04:XX,05:XX	04:XX,09:XX	02:02,08:XX	02:XX,03:XX	4/21	14/15b	4.14/21.15b
	452	08:XX,11:XX	04:XX,07:XX	04:XX,05:XX	04:03-04,10:XX	06:XX,07:XX	01:XX,03:XX	7/43	19a/23	7.23/43.19a
	453	08:XX,12:XX,13:01	05:XX,0601	10:XX,12:XX	04:XX,04:03-04	02:02,03:02-03	02:XX	22/35	13/15b	22.15b/35.13
	454	08:XX,16:03	04:XX,07:XX	05:XX,06:XX	04:03-04,10:XX	06:XX,07:XX	01:XX,03:XX	7/24	19a/23	7.23/24.19a

* One of the class I haplotypes of pig 464 may have been generated by recombination between SLA-1 of Lr-33.0 and SLA-2/SLA-3 of Lr-7.0 or 28.0, resulting in a recombinant haplotype as SLA-1*blank/undetected-SLA-3*07:XX–SLA-2*05:XX.

## Supplementary Materials

The following are available online at https://www.mdpi.com/2076-393X/8/2/234/s1, Figure S1: Adenovirus-driven expression of ASFV genes, Figure S2: Adenovirus driven expression of ASFV genes, Figure S3: MVA driven expression of ASFV genes, Figure S4: Infectious virus in the blood-stream of surviving pigs, Figure S5: Viral load in tissues, Figure S6: IgG specific for B602L and CP204L, Figure S7: Comparison of antigen-specific immune response between experiments 1 and 2, Figure S8: Antigen-specific IFNγ responses in animals from experiment 2, Figure S9: Scoring of macroscopic lesions in experiment 2.
