# Supplementary material for: A Pool of Eight Virally Vectored African Swine Fever Antigens Protect Pigs against Fatal Disease
Source: Vaccines (Basel). 2020 May 18;8(2):234. doi: 10.3390/vaccines8020234 (PMC7349991; doi:10.3390/vaccines8020234)
Supplement: Supplementary file 1 [file vaccines-08-00234-s001.pdf]

vaccines

MDPI

Article

Supplementary Material for a pool of eight virally vectored African swine fever antigens protect pigs against fatal disease:

Lynnette C. Goatley[1], Ana Luisa Reis[1], Raquel Portugal[1], Hannah Goldswain[1], Gareth L. Shimmon[1], Zoe Hargreaves[1], Chak-Sum Ho[2], María Montoya[1]‡, Pedro J. Sánchez-Cordón[1]†, Geraldine Taylor[1], Linda K. Dixon[1], Christopher L. Netherton[1]*

[1] The Pirbright Institute, Ash Road, Pirbright, Surrey, UK
[2] Gift of Hope Organ and Tissue Donor Network, Itasca, Illinois, USA

* Correspondence: christopher.netherton@pirbright.ac.uk; Tel.: +44(0)1483 232441.

† Present Address: Animal and Plant Health Agency, Addlestone, UK
‡ Present Address: Centro de Investigaciones Biológicas Margarita Salas (CIB-CSIC). Ramiro de Maeztu 9, Madrid 28040, Spain

Supplementary Methods

Protein expression analysis

Vero cells or porcine bone marrow cultures were infected with 125 IU/cell of rAd or 10 pfu/cell MVA encoding ASFV genes. Cells were fixed with 4% paraformaldehyde for 30 minutes or lysed in sample preparation buffer 20 hour post infection (hpi) with MVA and 48 hpi with rAd. Cells for immunofluorescence were permeabilised with 0.2% Triton X-100 in PBS, incubated with blocking buffer (50 mM Tris pH 7.4, 150 mM NaCl, 0.2% (w/v) gelatin, 10% (v/v) normal goat serum). Primary and secondary antibodies were also diluted in blocking buffer, cells were washed with PBS between all stages.

Protein lysates were resolved with 10% bis-tris gels, transferred to PVDF membranes, blocked with 5% milk powder and then probed overnight with primary antibody diluted in 5% BSA. Secondary antibodies were diluted in 5% milk powder, all solutions were based on TBS containing 0.2% Tween 20. Bands were detected by enhanced chemiluminescence (Pierce) with either X-ray film or with a Syngene G-box.

IgG specific sandwich ELISA

MaxiSorp 96 well plates were coated overnight at 2 - 8°C with recombinant CP204L and B602L (Reis et al., 2007) diluted in sodium carbonate buffer at 2 μg/ml. Plates were washed five times with PBS containing 0.2% tween 20 (PBS-T) and then blocked for one hour at 37°C with 5% milk powder diluted in PBS-T. Plated out sera diluted 1:100 in milk-PBS-T and incubated at room temperature for 5 hours with shaking. Washed five times with PBS-T and then incubated overnight with anti-porcine IgG diluted in milk-PBS-T overnight at 2 - 8°C. Finally, plates were washed again and developed with 3-dimethylaminobenzoic acid/3-methyl-2-benzothiazolinone hydrazine hydrochloride/H_2O_2 dissolved in 0.1 M phosphate buffer. After stopping the reaction with 3 M H_2SO_4 (50 μl per well), the absorbance at 620 nm was read on a Cytation3 microplate reader (Biotek).

Humane end-points

The following humane end-points were used in both experiments.

1. Pyrexia	a) The animal will be euthanized if it has a fever above 40.5°C and shows other clinical signs at the beginning of the third consecutive day.<br>b) If the animal has no other clinical signs, apart from a raised temperature, it will be euthanized at the beginning of the fourth consecutive day.
2. Behavior	The animal will be euthanized if it has a tendency to stay isolated, shows a delayed response to stimuli (gets up slowly when touched), and/or presents an abnormal posture e.g. head hung or back arched for two consecutive days.
3. Anorexia	The animal will be euthanized if it is not eating on a second consecutive day.
4. Digestive system	The animal will be euthanized if it has hemorrhagic diarrhea.
5. Respiratory system	Any pig showing an increase in breathing rate at the beginning of the second day without improvement will be euthanized at the beginning of the second day.
6. Lameness	All animals showing lameness will be treated with antibiotics and/or suitable analgesia at first signs.<br>a) Those animals showing non-weight bearing lameness will be euthanized 48 hours after treatment if it does not show improvement.<br>b) Those animals showing weight bearing lameness will be euthanized beginning of the fifth consecutive day after treatment if lameness has not improved significantly.<br>c) If there is a recurrence of the lameness, due to a regulated procedure, within 21 days from initial onset of lameness the animal will be euthanized on the same day.<br>d) If the animal becomes lame, due to a regulated procedure, more than 21 days from the first signs of initial lameness it will be treated as if it was the first instance as point one above.<br>e) If the animal becomes lame due to a regulated procedure a third time, it will be euthanized on the same day.
7.	Any animal showing three or more of the above clinical signs combined on a single day will be euthanized on the same day even if the duration of individual endpoints has not been reached.

Endpoint 7 was the most common reason for euthanasia (9 animals), followed by endpoints 1b and 3 (six animals each) and endpoint 2 (1 animal). No animals were euthanized for reaching endpoints 4, 5 or 6.

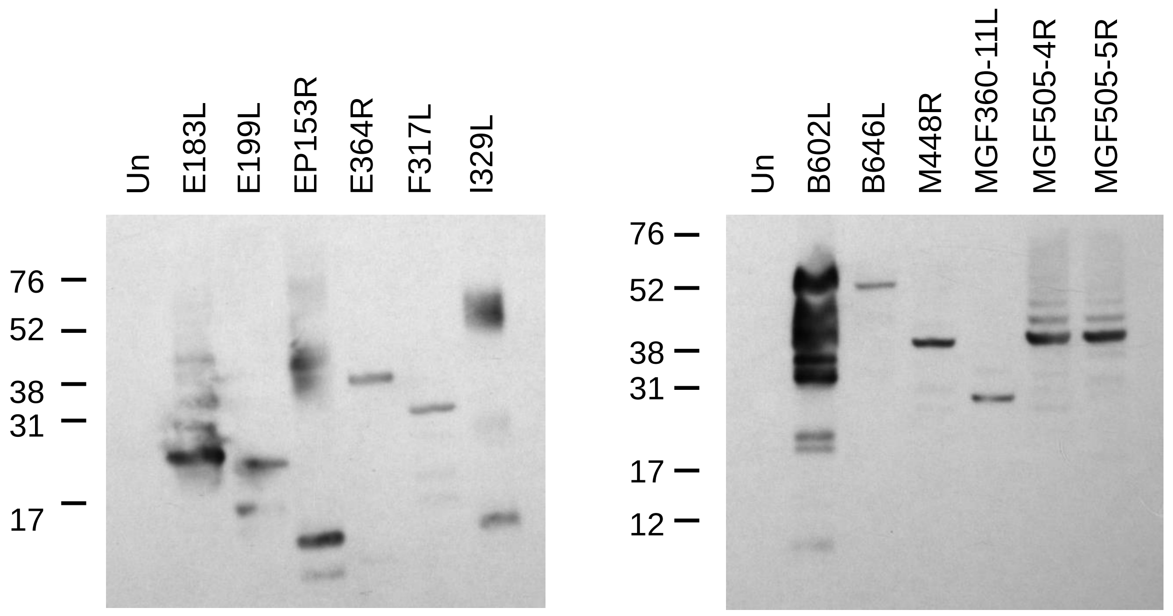

Figure S1: Adenovirus driven expression of ASFV genes. Cells were transduced with replication deficient human adenovirus 5 expressing the indicated ASFV genes and then lysed 48 hours later. Gene expression was determined by immunoblot using anti-HA clone 3F10.

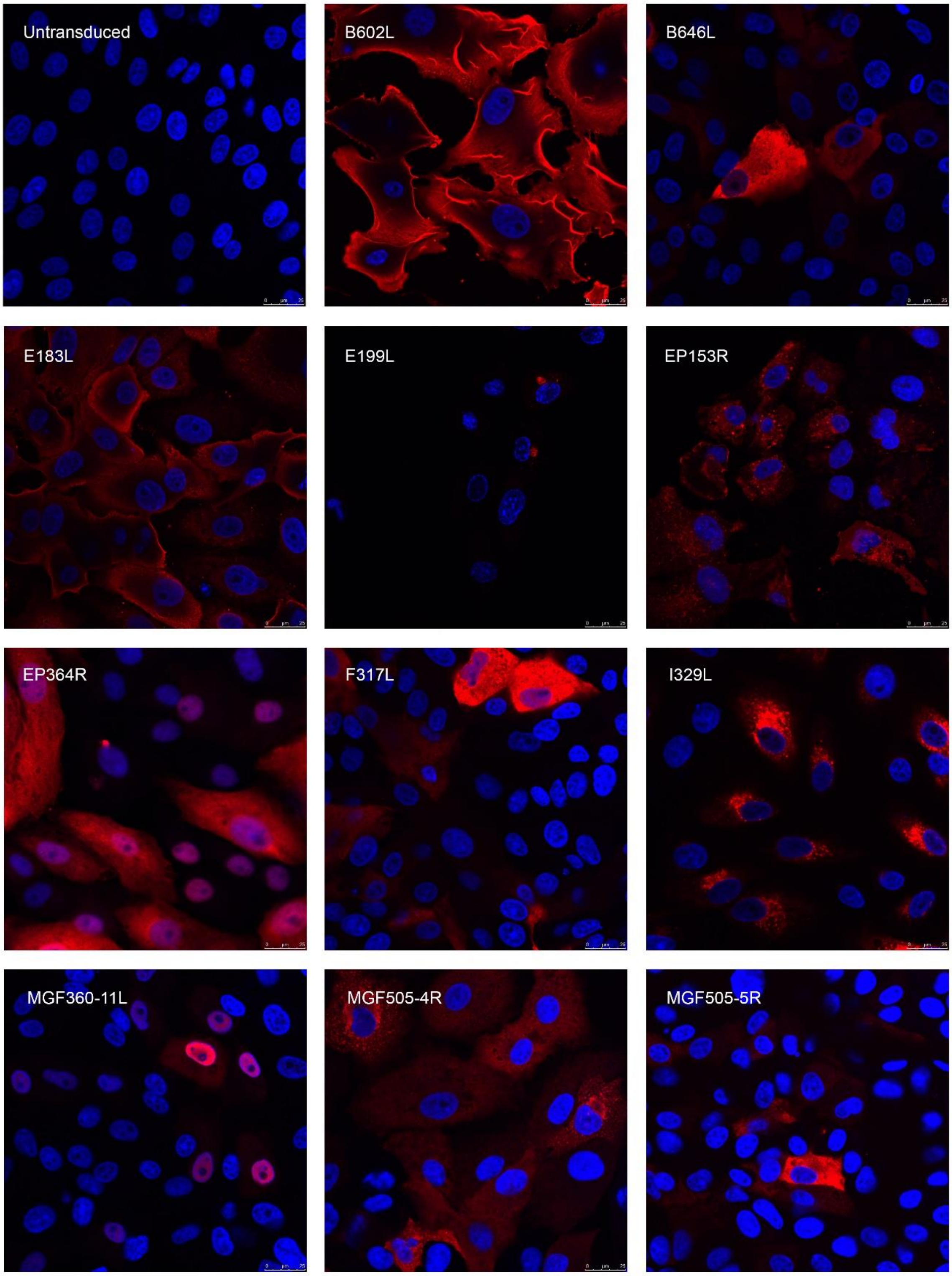

Figure S2: Adenovirus driven expression of ASFV genes. Cells were transduced with replication deficient human adenovirus 5 expressing the indicated ASFV genes and then or fixed 48 hours later. Gene expression was determined by indirect immunofluorescence using anti-HA clone 3F10.

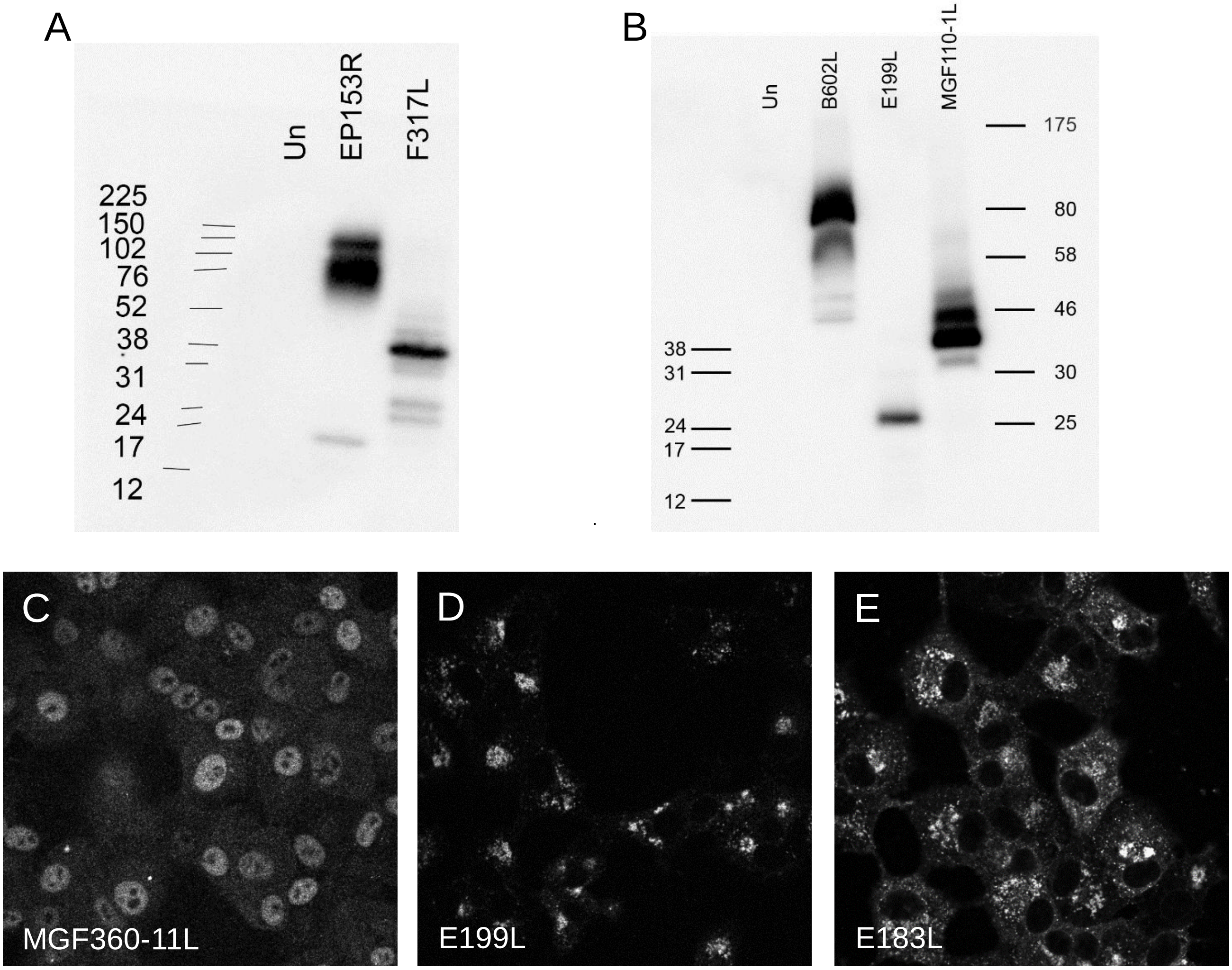

Figure S3: MVA driven expression of ASFV genes. Cells were infected with MVA expressing the indicated ASFV genes and then either lysed (Panel A, B) or fixed (C-E) 20 hours later. Gene expression was determined by immunoblot or indirect immunofluorescence using anti-HA clone 3F10 (A-D) or anti-E183L RB7 (E).

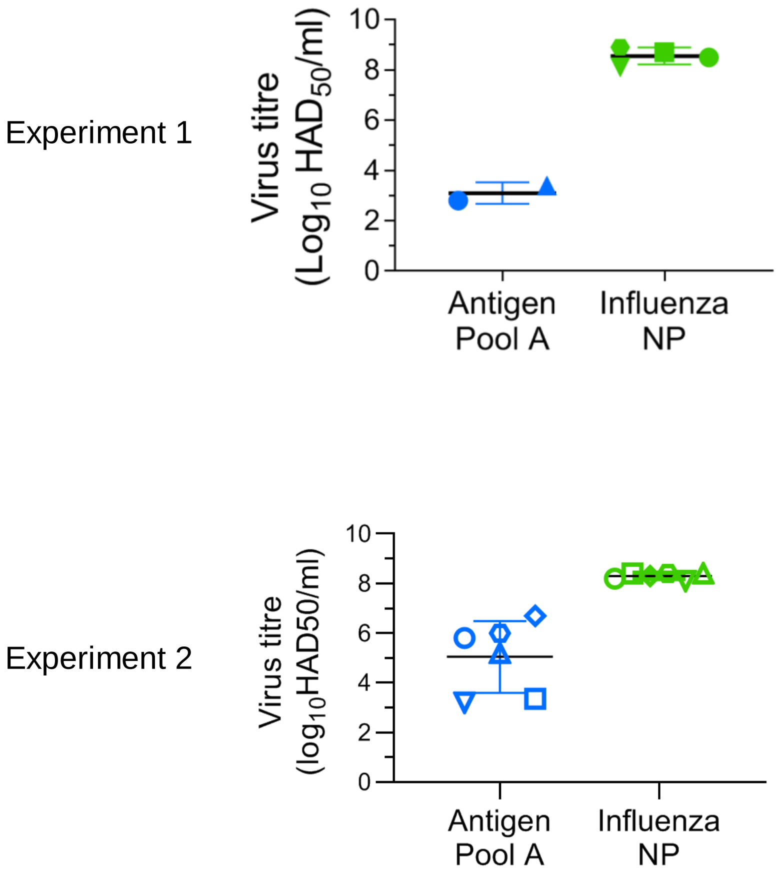

Figure S4: Infectious virus in the blood stream of surviving pigs. Blood taken from surviving animals in experiment 1 (pigs 264 and 266) and experiment 2 (461 to 465) twenty days post challenge as well as selected animals from the controls for Experiment 1 (270, 271, 273 & 275) and 2 (455 to 460) taken four to six days post challenge were tested for the presence of live virus by hemadsorption assay. Black horizontal bars indicate the means and the coloured error bars show the standard deviation from that mean. Asterisks indicate significant differences (Welch's T test, $t = 5.481$, $df = 5.076$) between pigs immunised with Antigen Pool A and Influenza NP ($p = 0.0026$).

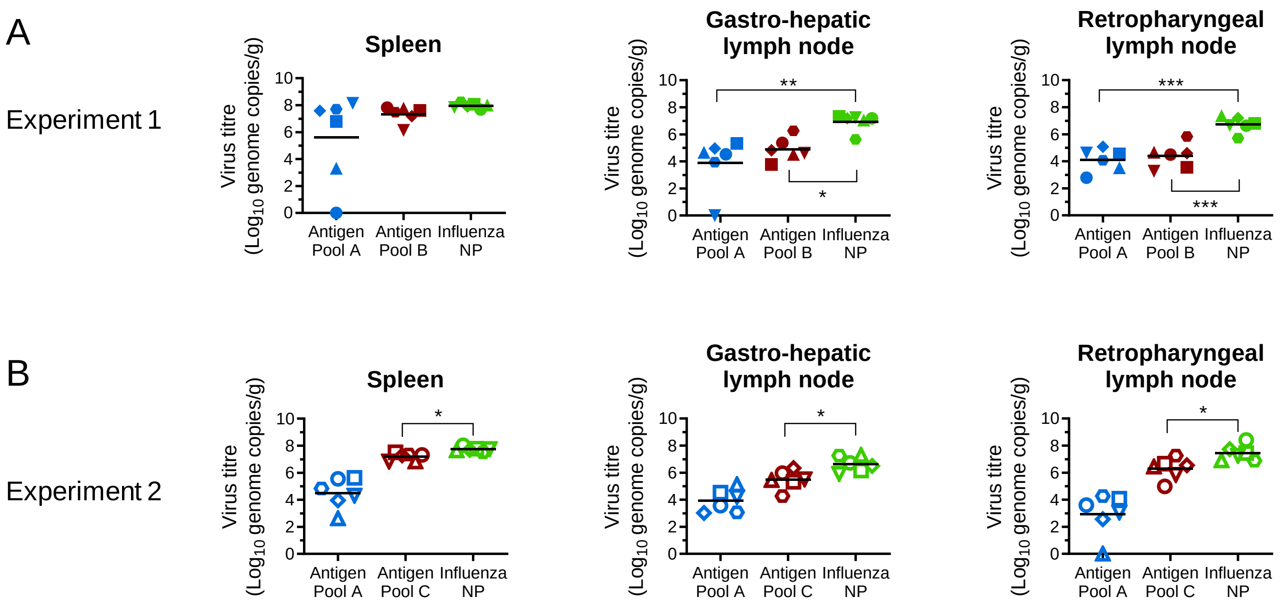

Figure S5: Viral load in tissues. Spleen, gastro-hepatic lymph node and retropharyngeal lymph node taken post mortem from Experiments 1 and 2 were extracted in duplicate and the viral titre determined by qPCR, with each extraction tested in duplicate. **A)** For experiment 1, tissues were collected four to six days post challenge except for pigs 264 and 266 (●,▲ respectively) which were collected twenty days post challenge. Samples from 264 and 266 were not included in statistical analysis and are shown for reference only. Bars indicate the mean of each group and asterisks indicate significant differences (ordinary one-way ANOVA) between Control and either of the antigen pools, * $p \leq 0.05$, ** $p \leq 0.01$, or *** $p \leq 0.001$. **B)** Experiment 2 tissues were collected from pigs immunised with Antigen Pool C and Influenza NP six days post challenge and from pigs immunised with Antigen Pool A twenty days post challenge. Data points are the mean of the duplicate extractions. Bars indicate the mean of each group and asterisks indicate significant differences (Welch's t-test) between the group immunised with influenza NP and Antigen Pool C, * $p \leq 0.05$.

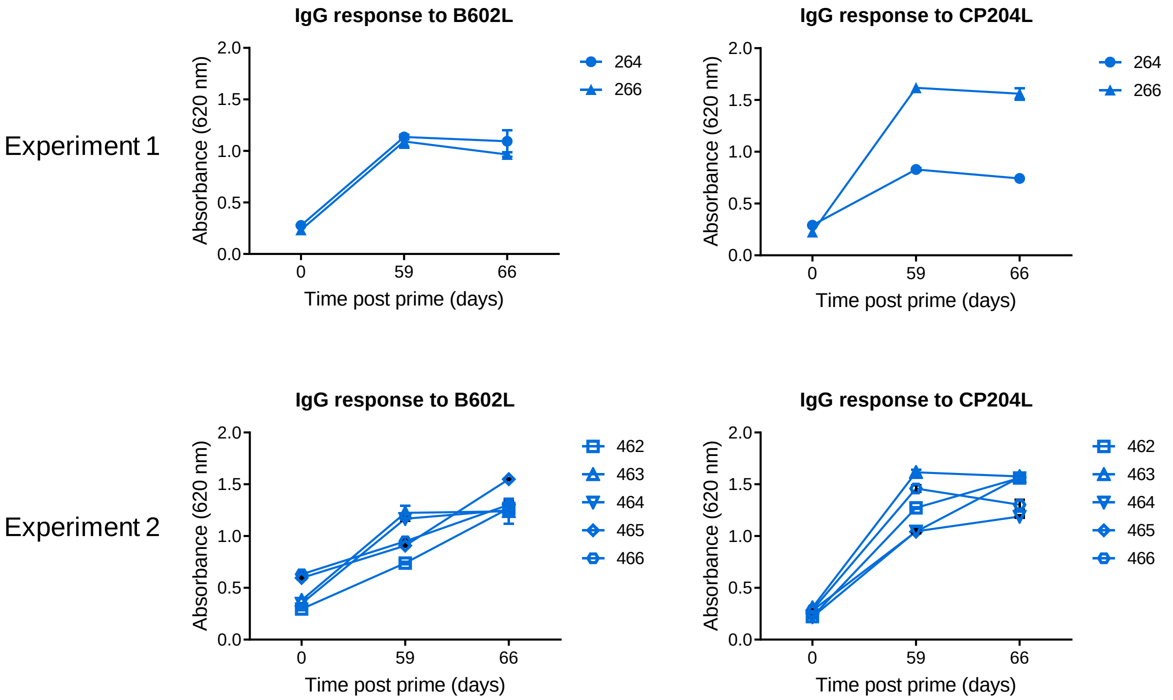

Figure S6: IgG specific for B602L and CP204L in protected animals. Sera from taken from surviving animals pre-prime (day 0), pre-challenge (day 59) and seven days post challenge (day 66) were analysed using IgG specific ELISAs against B602L and CP204L (See Supplemental Methods). Individual data points represent the mean of duplicate wells and error bars indicated the standard deviation. No sera from pig 461 seven days post challenge was available and therefore this animal was excluded from this analysis. There was no difference in the means of the response to either B602L and CP204L between day 59 and day 66 in experiment 2 (RM one-way ANOVA with Tukey's multiple comparison test, $p = 0.1156$; F (1.307, 5.229) = 36.74 and $p = 0.4816$; F (1.610, 6.439) = 80.02 respectively).

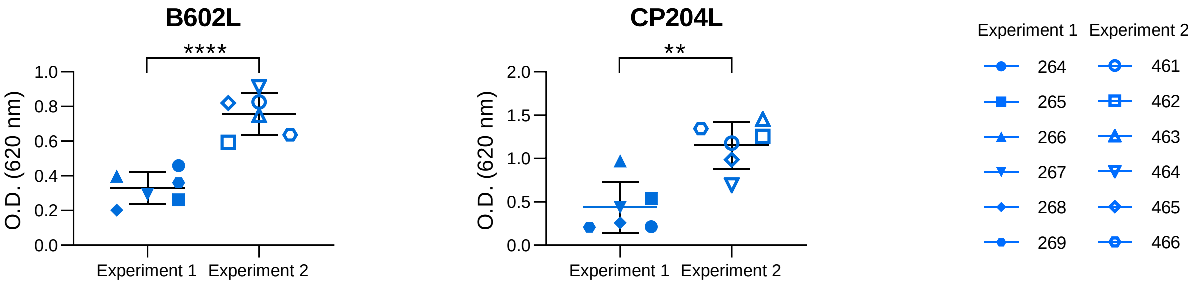

Figure S7: Comparison of antigen specific immune responses between Experiments 1 and 2. Sera from taken from animals pre-challenge (day 59) were analysed using B602L and CP204L ELISAs. Individual data points represent the mean of duplicate wells and error bars indicated the standard deviation. This data is taken from Figure 1C and 5B (B602L) and Figure 1E, 5C (CP204L). Differences in the means of the two experiments were analysed using Welch's t-test, **** $p \leq 0.0001$, ** $p = 0.0015$.

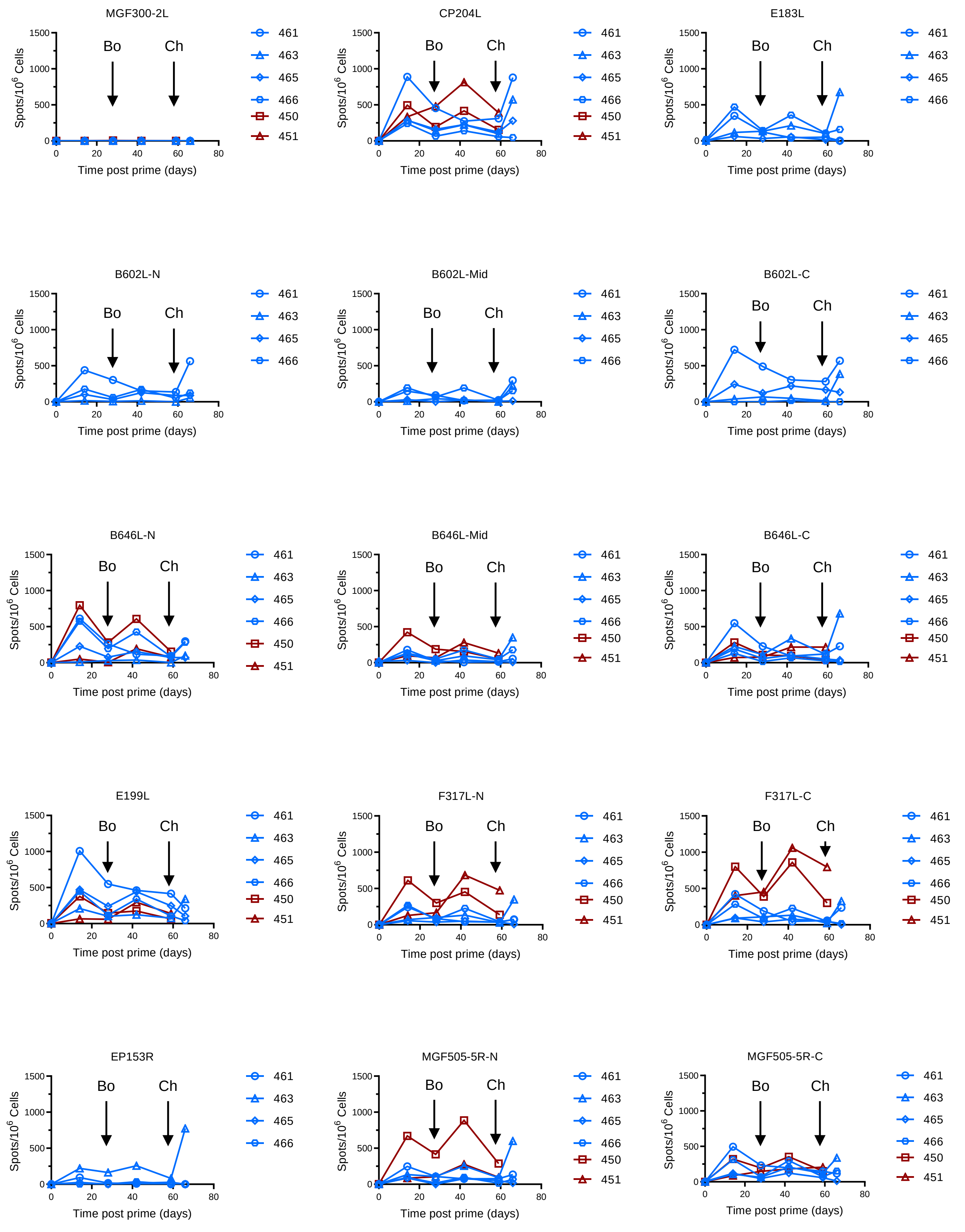

Figure S8: Antigen specific IFNγ responses in animals from Experiment 2 immunised with Antigen Pool A (blue) or Antigen Pool B (red). The number of IFNγ-producing PBMC stimulated by pools of peptides were enumerated by ELIspot on the indicated days post prime and subject to background subtraction. N, mid and C indicate pools of peptides that correspond to the N-terminus, mid-section or C-terminus of the proteins encoded by B602L, B646L, F317L and MGF505-5R. Timing of boost (Bo) and challenge (Ch) are indicated.

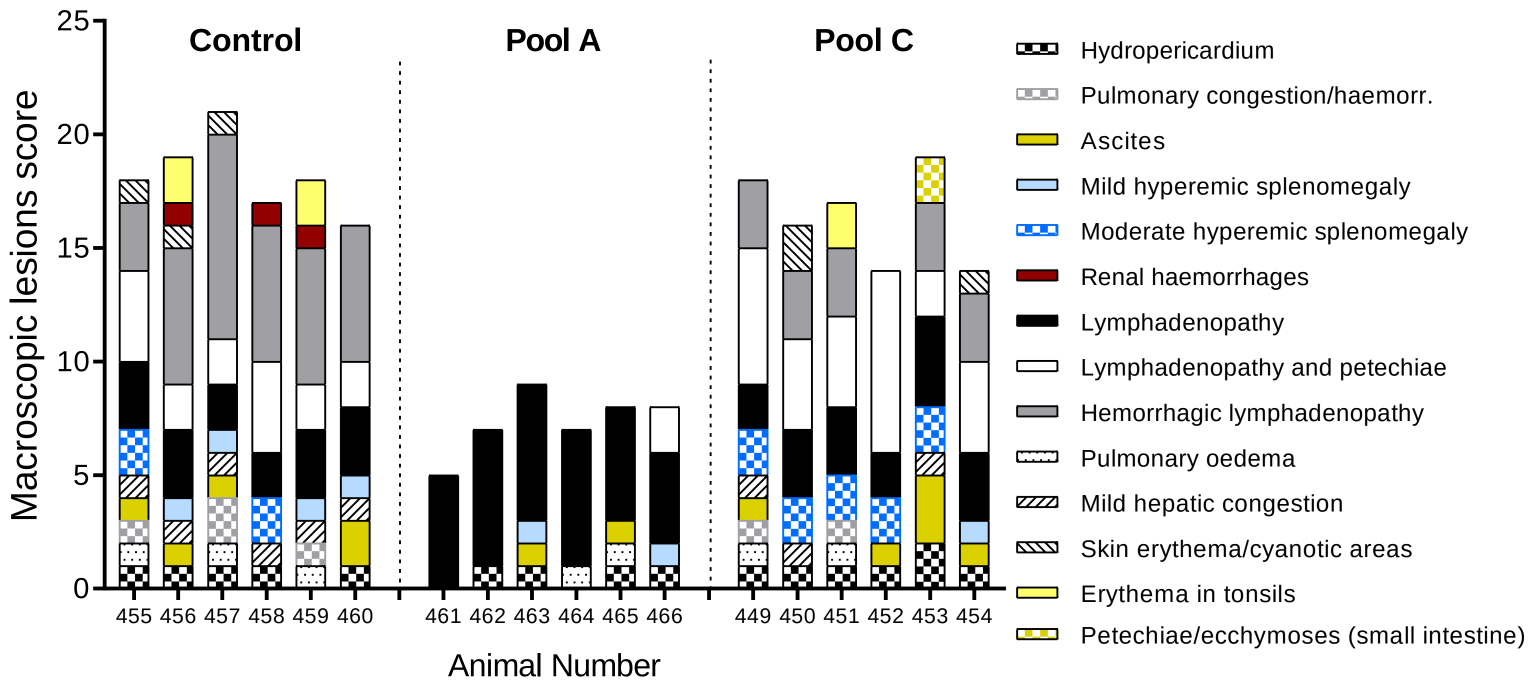

Figure S9: Scoring of macroscopic lesions in Experiment 2. Macroscopic lesions were evaluated as described by Galindo-Cardiel *et al.*, 2013 [44]. The tissues evaluated and the gross lesions observed are indicated on the graph by different patterns and/or colours. All of the animals in the control group and those immunised with Antigen Pool C were euthanized six days post challenge. The animals immunised with Antigen Pool A were euthanized twenty days post challenge.
